# Agonistic GITR treatment enhances antitumor immune responses and suppresses tumor progression in pancreatic ductal adenocarcinoma

**DOI:** 10.1007/s00535-026-02347-y

**Published:** 2026-02-01

**Authors:** Steve Robatel, Hanne Hillen, Ivanina Mutisheva, Joshua C. Müller, Martin Wartenberg, Feiyang Ma, Lukas Bäriswyl, Jef Evenepoel, Colinda L. G. J. Scheele, Delphine J. Lee, Robert L. Modlin, Ulf Kessler, Max Nobis, Kaspar Z’graggen, Mirjam Schenk

**Affiliations:** 1 Division of Experimental Pathology, Institute of Tissue Medicine and Pathology (ITMP), University of Bern, Bern, Switzerland; 2 Graduate School Cellular and Biomedical Sciences, University of Bern, Bern, Switzerland; 3 Laboratory for Intravital Imaging and Dynamics of Tumor Progression, Department of Oncology, VIB-KU Leuven Center for Cancer Biology, Louvain, Belgium; 4 Division of Surgical Pathology, Institute of Tissue Medicine and Pathology (ITMP), University of Bern, Bern, Switzerland; 5 Department of Cell and Developmental Biology, Feinberg School of Medicine, Northwestern University, Chicago, IL 60611 USA; 6 Laboratory for Enteric Neuroscience (LENS), Translational Research Center for Gastrointestinal Disorders (TARGID), KU Leuven, Louvain, Belgium; 7 The Lundquist Institute for Biomedical Innovation, Torrance, CA USA; 8 Division of Dermatology, Department of Medicine, Harbor-UCLA Medical Center, Torrance, CA USA; 9 David Geffen School of Medicine, University of California Los Angeles, Los Angeles, CA USA; 10 Division of Dermatology, School of Medicine, University of California Los Angeles, Los Angeles, CA USA; 11 Department of Pediatric Surgery, Inselspital, Bern University Hospital and University of Bern, Bern, Switzerland; 12 Manchester Cell-Matrix Centre, Division of Cell Matrix Biology and Regenerative Medicine, School of Biological Sciences, Faculty of Biology Medicine and Health, The University of Manchester, Manchester, UK; 13 Swiss Pancreas Clinic, Bern, Switzerland; 14 Christine Kühne—Center for Allergy Research and Education (CK-CARE), Davos, Switzerland

**Keywords:** Pancreatic ductal adenocarcinoma, Tumor microenvironment, Immunotherapy, Immunomodulation, Transcriptomics

## Abstract

**Background:**

Despite advances, immunotherapy has shown limited efficacy in pancreatic ductal adenocarcinoma (PDAC). The profoundly immunosuppressive tumor microenvironment (TME) of PDAC restricts effective antitumor immune responses, necessitating the development of novel therapeutic approaches. Emerging evidence suggests that modulating the TME could enhance immunotherapy outcomes, with glucocorticoid-induced TNFR-related protein (GITR) presenting as a promising target.

**Methods:**

We performed in vivo studies using the Pan02 mouse model of PDAC, where we activated GITR. Complementary analyses were performed on human PDAC samples that were obtained from surgical resections, both from treatment-naive patients and those undergoing neoadjuvant chemotherapy. Human PDAC samples were assessed using scRNA-seq, spatial transcriptomics, and immunofluorescence.

**Results:**

GITR was found to be significantly overexpressed in PDAC tissues compared to normal adjacent pancreatic tissue, with further upregulation observed following neoadjuvant chemotherapy. These findings were corroborated in Pan02 mouse model. GITR activation in vivo led to a reduction in regulatory T cells (Tregs) and an increase in activated cytotoxic effector cells within the TME, resulting in suppressed tumor growth and extended survival. Spatial transcriptomic analysis revealed that GITR expression was predominantly localized to lymphocytes in close proximity to tumor cells in human PDAC. Additionally, long-term survival PDAC patients showed high levels of GITR^+^ lymphocytes, underscoring its clinical relevance.

**Conclusions:**

This study identifies GITR as a key regulator of the immunosuppressive TME in PDAC. By promoting T cell activation and effector functions, GITR represents a promising target for immunotherapeutic treatment in PDAC. Combining GITR activation with standard chemotherapy may offer a promising strategy to improve outcomes for PDAC patients.

**Graphical abstract:**

**Figure:**
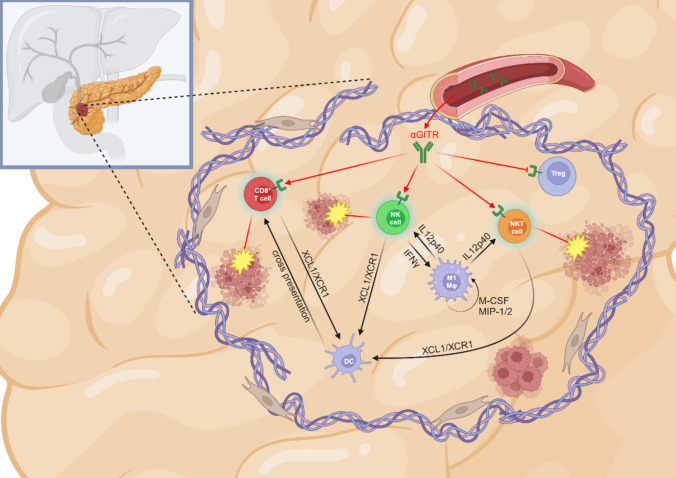
Mechanistic Insights into GITR Activation in PDAC. In mouse pancreatic ductal adenocarcinoma (PDAC), systemic agonistic GITR therapy reprograms the tumor microenvironment (TME) by shifting its immune composition from an immunosuppressive state to a more immune-activated and cytotoxic milieu. This shift is mediated through key pathways involving IL-12p40, M-CSF, MIP-1/2, and XCL1/XCR1 signaling.

**Supplementary Information:**

The online version contains supplementary material available at 10.1007/s00535-026-02347-y.

## Introduction

Pancreatic ductal adenocarcinoma (PDAC) represents the most common form (up to 90%) of pancreatic cancer. PDAC is known to be one of the most aggressive cancers due to its rapid progression, resistance to therapies, and the dissemination of micrometastases, which significantly reduce patient survival [1–3]. These characteristics are reflected in a poor 5 year overall survival (OS) rate of approximately 13%. Furthermore, in the majority of PDAC cases, the cancer has either metastasized to distant sites (50%) or advanced locally (30%), and only about 20% of PDAC are considered candidates for primary resection at the time of diagnosis. For these patients, surgery is the only treatment option with curative intent [4]. In patients with locally advanced cancer, neoadjuvant treatment followed by resection is a novel and curative approach, but tumors with distant metastasis are treated palliatively according to current standards. Thus, there is an urgent need for the development of new therapeutic strategies.

A major advance in PDAC treatment was the establishment of the chemotherapy FOLFIRINOX (folinic acid, fluorouracil, irinotecan and oxaliplatin), initially for the treatment of metastatic PDAC, then in the adjuvant setting, where it has shown to significantly extend patient survival compared to the previous standard chemotherapeutic agent Gemcitabine [5, 6]. Recently, FOLFIRINOX has also shown promising results as a neoadjuvant therapy, improving resectability and patient survival [7] with the disadvantage of more severe toxic side effects [8]. Therefore, patients with poor physical and medical conditions are usually treated with a combination of gemcitabine and nab-paclitaxel. It has been shown that neoadjuvant chemotherapy (CTx) can alter the immune compartment of the TME in PDAC [9–11] and the characterization of these immunologic alterations may facilitate the identification of novel therapeutic combinations.

Over the past 2 decades, immune checkpoint inhibitors, particularly treatments targeting programmed cell death protein 1 (PD-1)/PD-1 ligand (PD-L1) and cytotoxic T-lymphocyte-associated protein 4 (CTLA-4), have shown remarkable efficacy in various cancers such as lung cancer, renal cell carcinoma, and melanoma [12–15]. Unfortunately, this could not be transferred to PDAC [12, 16]. The reasons for this are manifold, but a major obstacle is the complex desmoplastic and immunosuppressive tumor microenvironment (TME). Studies have shown that CD4^+^FoxP3^+^ regulatory T cells (Tregs) are critical inhibitors of antitumor immune responses and therefore represent an attractive target for the development of novel immunotherapies in pancreatic cancer. In mouse models of pancreatic cancer, their depletion has already been shown to lead to increased activation of CD8^+^ T cells, delayed tumor growth, and prolonged survival time [17].

The type I transmembrane protein glucocorticoid-induced TNFR-related protein (GITR), is a costimulatory receptor that is constitutively expressed at high levels on activated T cells, Tregs and NK cells, and at lower levels on other lymphocytes [18, 19]. GITR activation leads to inhibition of Treg suppressive function [20] and confers resistance of effector T cells to Treg suppression [21]. Similar observations have been made in NK cells [19]. While many features of GITR are conserved between humans and mice, there are some differences. The expression of GITR is largely similar across both species [18, 19], suggesting a conserved expression pattern. In mice, GITR plays a key role in the differentiation of thymic Tregs (tTregs) [22] and the overall expansion of the Treg population [23]. In humans, while GITR is also involved in Treg activation, it serves as a reliable marker for certain Treg subsets, specifically those that are FoxP3 and CD25 low but GITR high [24]. Although similar effects of GITR activation have been observed in human Tregs from certain solid tumors, the extent of these effects appear less definitive than in mice and varies depending on the tumor type [23]. In recent years, several GITR agonistic agents have been tested in clinical trials for advanced solid tumors and demonstrated an acceptable safety profile [25]. However, these clinical trials included too few patients with PDAC to provide conclusive results on the efficacy of GITR therapy for PDAC.

By comparing the expression of key immunoregulatory molecules (e.g., PD-L1, PD1, CTLA4, etc.) in human PDAC and normal adjacent pancreatic tissue, we found that specifically GITR is highly expressed in PDAC and is even further induced in tumors from patients receiving CTx. Thus, we hypothesize that targeting immunostimulatory molecules, in particular agonistic treatment with αGITR monoclonal antibody (mAb), could be an effective complementary treatment for PDAC.

## Materials and methods

### *Mice, tumor inoculation, and *in vivo* studies*

C57BL/6 mice were received from Janvier Labs (France), and experimental animals were bred and housed in specific pathogen-free conditions in the central animal facility (CAF). All animal experiments were performed in accordance with federal regulations and approved by the cantonal veterinary office (BE23/2022). Eight to 12 week-old age and sex-matched animals were used for all experiments. Mice were randomly assigned to different treatment groups prior to tumor injection. On day 0, tumors were engrafted by subcutaneous (s.c.) injection of 2 × 10^5^ Pan02 cells onto the left flanks of the mice in 100 µl PBS [26, 27]. Mice were treated in a random order, i.e., regardless of the treatment group, with intraperitoneal (i.p.) injection of PBS, agonistic anti-mouse GITR monoclonal antibody (500 μg in 200 μL i.p.) (clone DTA-1, InVivoMab). Depletion of NK cells was achieved using anti-mouse NK1.1 mAb (200 μg in 200 μL i.p.) (clone PK136, InVivoMab) and of CD8^+^ T cells using anti-mouse CD8α mAb (200 μg in 200 μL i.p.) (clone 53–6.7, InVivoMab). FOLFIRINOX was prepared to a working concentration of 1 mg calcium folinate (Sigma), 0.5mg 5-Fu (Sigma), 0.01 mg oxaliplatin (Sigma) and 0.1 mg irinotecan (Sandoz) in 50 μL PBS and injected intratumorally. Tumor growth was monitored in a blinded manner, i.e. before knowing if each mouse was to receive the treatment or the control, by measuring 2 dimensions using a digital caliper. Tumor volume was calculated using the following formula *V* = (length*width^2^)/2. For the survival experiment, mice were euthanized when tumor size reached 500 mm^3^ in accordance with humane endpoint of our protocols. On day 14 post-tumor-inoculation, mice were euthanized, and the tumors were isolated.

### Processing of clinical samples

Human PDAC samples were collected in accordance with guidelines of the cantonal ethics committee (KEK) in Bern under approved protocols (KEK ID: 2017–02246). All participants provided written general consent. Tissue samples were obtained from the Beau-Site Hirslanden Klinik in Bern, Switzerland and processed immediately after collection. Fat tissue was removed from the samples and tumors were cut into 2–4 mm pieces and placed into a gentleMACS C tube (Miltenyi). Each sample was enzymatically digested using the tumor Dissociation kit and the gentleMACS™ Dissociator according to the manufacturer’s protocol (Miltenyi). Cells were filtered through a 70 µm cell strainer to discard any remaining undigested tissue before proceeding with the flow cytometric staining. To limit the batch effect between samples and to comply with surgery schedules, samples were crosslinked for 30 min using DSP and stored for 24 h, following our recently published protocol for living cells preservation for scRNA-seq [28].

### PDAC tissue micro array (TMA) dataset

The PDAC TMA was constructed at the translational research unit (TRU) platform of the institute of tissue medicine and pathology (ITMP). This TMA contains cancer tissue of 117 PDAC cases (50 female, 67 male) resected in a curative setting at the department of Visceral Surgery of Inselspital Bern and diagnosed at ITMP between the years 2014 and 2020. The study followed the guidelines of the world medical association declaration of Helsinki 1964, updated in October 2013, and was conducted after approval by the KEK of Bern (CEC ID2020-00498). All participants provided written general consent. The TMA contained 3 spots from each case (tumor front, tumor center, tumor stroma), leading to a total number of 351 tissue spots. Thirteen of these 117 cases were treated with neoadjuvant chemotherapy followed by surgical resection, and the majority of the cases (104) were resected curatively. All cases were comprehensively characterized clinico-pathologically, including tumor, node, and metastasis (TNM) stage. On pathologic examination, all cases were tumor stage I, II or III cases, according to the union for international cancer control (UICC) TNM classification of malignant tumors, 8th edition [29].

### Eve technologies discovery assay

Tissue samples were snap frozen in liquid nitrogen and stored at −80 °C. Tissue was homogenized using the tissuelyser with beads (Qiagen) and sample protein concentration was normalized according to the Eve technologies sample preparation guide. Samples were then shipped on dry ice, and we performed the human immuno-oncology checkpoint 16-plex assay and the mouse cytokine/chemokine 44-plex discovery (eve technologies).

### Fluorescence-activated cell sorting (FACS) and flow cytometric analysis

Zombie aqua viability dye (zombie dye, Biolegend) was used to discriminate dead cells. For the experiments with the mouse model, the following antibodies were used: PE-Cy7 anti-CD45.2 (clone 104, Biolegend), FITC anti-CD3ε (clone 145-2C11, Biolegend), APC-Cy7 anti-CD4 (clone RM4-5, Biolegend), BV785 anti-CD8 (clone 53–6.7, Biolegend), AF647 anti-FoxP3 (clone MF-14, Biolegend), PE anti-NK1.1 (clone PK136, Biolegend), AF700 anti-F4/80 (clone BM8, Biolegend), FITC anti-CD11c (clone N418, Biolegend), BV785 anti-CD11b (clone M1/70), BV421 anti-MHCII (clone M5/114.15.2, Biolegend), PE anti-CD357 (GITR) (clone YGITR765, Biolegend). For human experiments, the following antibodies were used: BV785 anti-CD45 (clone HI30, Biolegend), BV421 anti-CD3 (clone OKT3, Biolegend), AF488 anti-CD56 (clone MEM188, Biolegend), PE anti-CD357 (GITR) (clone 108–17, Biolegend). Samples were analyzed using a Beckman Coulter Cytoflex S and FACS before scRNA-seq was performed using a Beckman Coulter MoFlo ASTRIOS BSL-2 cell sorter at the FACS facility, department of biomedical research (DBMR), university of Bern, Bern, Switzerland. FlowJo (V10.8.1) was used for flow cytometric data analysis.

### *10* × *genomics illumina scRNA-Seq*

The samples were dissociated and sorted for CD45^+^ cells using the above-described protocols. Samples for scRNA-seq were submitted to the next generation sequencing (NGS) platform at the university of Bern. They performed gel bead-in-emulsion generation, quality control (QC), barcoding of the cells (performed on a chromium controller, Single Cell 3′ v3), and finally the sequencing on an illumina NovaSeq 6000 sequencer, according to the 10X manufacturer protocol.

### scRNA-seq data analysis

Raw reads were aligned using a pre-built reference for mouse and human genomes, refdata-gex-mm10-2020-A; mouse reference, mm10 (GENCODE vM23/Ensembl 98) and refdata-gex-GRCh38-2020-A; human reference, GRCh38 (GENCODE v23/Ensembl 98) using CellRanger (version 7.1, 10 × Genomics Inc., Pleasanton, CA, USA) [30]. The confirmation scRNA-seq dataset from Werba, Weissinger et al. [31] was downloaded from GEO with the accession number GSE205013. All downstream analyses, including QC were performed using the Seurat (version 4.3.0) [32] and integration was performed using Harmony (version 0.1.1) [33] packages in R (version 4.2.2) as previously described [34]. For cluster annotation, the consensus of multiple methods was used. *ImGenData and MouseRNAseqData* libraries from the Singler package (version 2.0.0) were used to annotate the clusters in the mouse dataset, and the *HumanPrimaryCellAtlasData, BlueprintEncodeData, NovershternHematopoieticData, MonacoImmuneData, DatabaseImmuneCellExpressionData* libraries were used for the human cluster identification as described previously [35]. The database of CIBERSORTx [36] was also used to identify the clusters. The differentially expressed cluster marker genes were extracted using the Seurat and SeuratExtend package and applied to the gene set enrichment analysis tool Enrichr to annotate the clusters [37–40]. Gene ontology was performed using the gene ontology analysis tool [41–43] and the ClueGo plug-in from the cytoscape software (V3.9.0) [44, 45]. CellChatDB (version 1.6.1) was applied for ligand-receptor analysis as described by Jin *et. al.* [46].

### Spatial transcriptomics

Clinical samples from PDAC surgeries were obtained and processed for spatial transcriptomics as follows. The sample was cut into a square shape of 8 × 8 mm and embedded in O.C.T. compound (Sakura) by freezing on dry ice and stored at −80 °C. Then each tissue was sectioned using a cryotome and placed on a visium spatial slide from 10x, fixed and stained for nuclei with DAPI (ThermoFisher) and CD45 (LCA, clone 2B11 + PD7/26, Agilent). The subsequent steps of imaging, permeabilization, cDNA synthesis and library construction were performed by following the 10 × spatial transcriptomics protocol. The raw sequencing data were then aligned to the pre-built reference for the human genome (refdata-gex-GRCh38-2020-A; human reference, GRCh38 (GENCODE v23/Ensembl 98)) by using space ranger (version 2.0.0, 10 × Genomics). Subsequent analysis and integration with scRNA-seq data were performed using the Seurat package (version 4.3.0) in R and following the workflow of the Satija Lab [49].

### Immunohistochemistry

Tissue and TMA sections were processed following the step by step IHC protocol (single analyte) from Akoya and using the Opal 3-Plex manual detection kit (NEL810001KT, Akoya BioSciences). The steps of blocking, target retrieval (Dako, #S2367), primary antibody incubation, introduction of HRP, signal amplification and antibody stripping were repeated for each analyte, namely OPAL690 anti-human CD45 (clone 2B11 + PD7/26, DAKO), OPAL570 anti-human CD3 (clone LN10, Leica), OPAL520 anti-human GITR (clone CAL52, Abcam), OPAL520 anti-human Pan-CK (NB600-579, NovusBio), OPAL570 anti-human FOXP3 (EP340, Bio SB), OPAL690 anti-human CD11b (EPR1344), OPAL520 anti-mouse CK-8 (ab53280), and OPAL570 anti-mouse Foxp3 (ab75763). Images were acquired using a panoramic 250 slide scanner from 3D Histech. Data was analyzed using the phenoplex workflow implemented in VisioPharm (version 2023.01).

### Statistical analysis

GraphPad Prism version 9.0 (GraphPad software version 9.0) or R were used for statistical analyses. The Mantel-Cox log-rank test was used to test for significant survival differences. For comparison of the means of 2 independent groups, unpaired *t* tests were used. Outlier tests were performed with default settings in GraphPad Prism and statistical significance was determined as follows: *p* ≤ 0.05*; *p* ≤ 0.01**; *p* ≤ 0.001***; *p* ≤ 0.0001****. Correction for multiple testing was performed using the Benjamini–Hochberg method.

## Results

### GITR is overexpressed in human PDAC and in vivo agonistic GITR treatment suppresses tumor growth

**Fig. 1:**
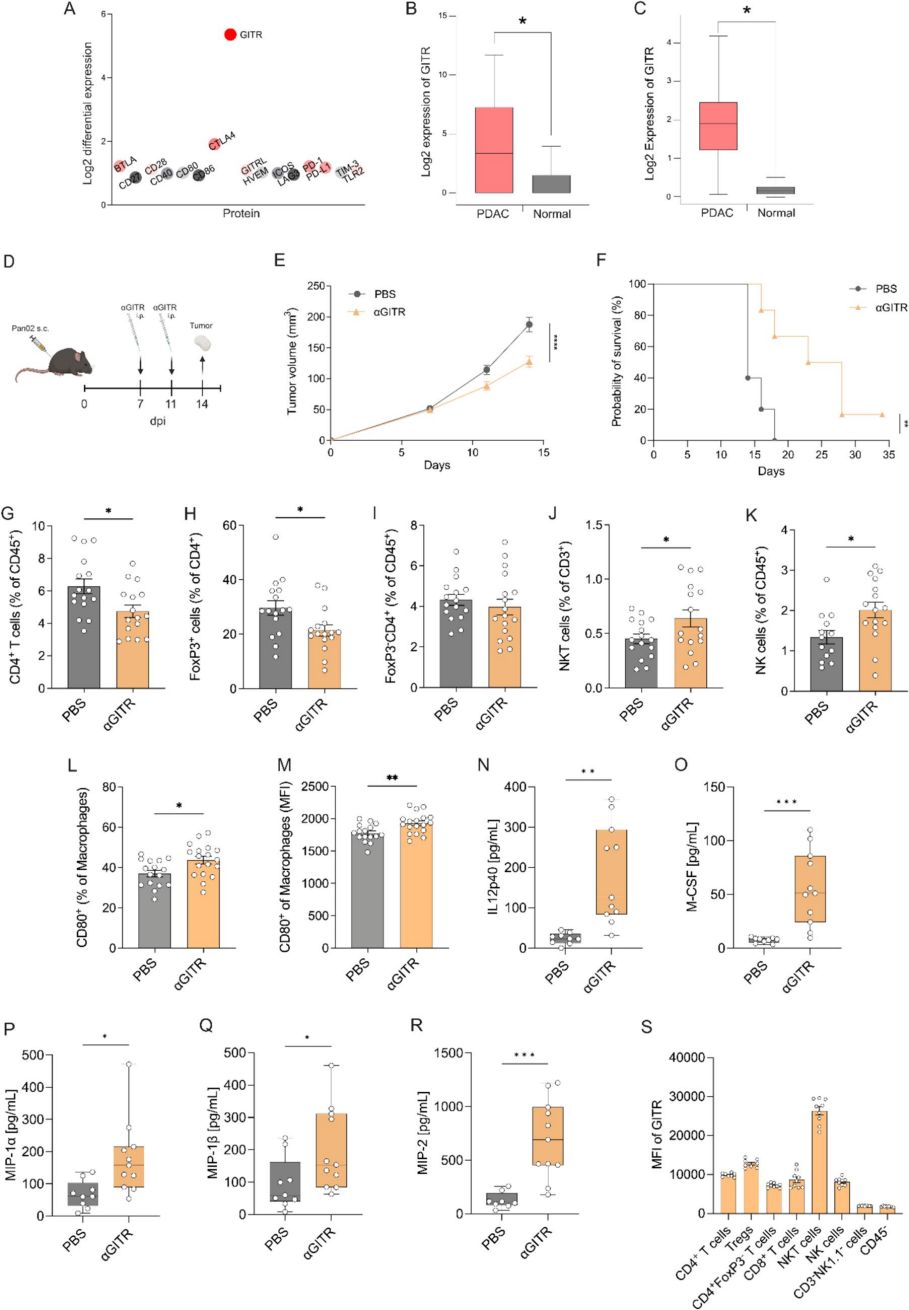
GITR is overexpressed in human PDAC and agonistic GITR treatment reduces tumor growth and Tregs and increases the number of tumor-infiltrating effector cells. **A** Log2 differential expression of proteins between PDAC and normal adjacent pancreas as measured by Eve technologies analysis. The panel contained BTLA, CD27, CD28, CD40, CD80, CD86, CTLA4, GITR, GITRL, HVEM, ICOS, LAG3, PD-1, PD-L1, TIM-3 and TLR2. Log2 expression of GITR protein [pg/mL] in human PDAC compared to normal adjacent pancreas, *n* = 15. **B**, **C** Log2 expression of GITR mRNA in (**B**) human PDAC compared to normal adjacent pancreas samples and (**C**) human PDAC compared to normal pancreas samples from the TCGA PAAD cohort, *n* = 179 (PDAC), *n* = 171 (Normal). **D** Graphical representation of the experimental design; Mice were injected s.c. on day 0 with Pan02 tumor cells and i.p. injection of αGITR antibody was performed at 7 and 11 days post-injection (dpi). Tumors were isolated on day 14. Tumor growth was measured on days 7, 11 and 14. **E** Tumor growth of mice treated with either αGITR antibody or PBS, *n* = 38. **F** Kaplan–Meier survival analysis of mice treated with αGITR antibody or PBS, *n* = 6. Mantel-Cox log-rank test. **G**–**M** flow cytometric analysis of isolated tumors stained with antibodies against (**G**) CD4, (**H**–**I**) FoxP3, (**J**) CD3, (**J**–**K**) NK1.1 and (**L**–**M**) CD80, *n* = 32. Observed protein concentration of (**N**) IL12p40, (**O**) M-CSF, (**P**) MIP-1α, (**Q**) MIP-1β and (**R**) MIP-2, n ≥ 9. **S** MFI of GITR on relevant immune cells from untreated tumors. Error bars show SEM, whiskers show minimum and maximum. Statistical significance was calculated using unpaired *t* test; *p* < 0.05*; *p* < 0.01**; *p* < 0.001***; *p* < 0.0001****

To identify potential immunotherapeutic candidates that are highly expressed in PDAC, we performed comparative proteomics analysis of 15 PDAC tissue and matched normal adjacent pancreas obtained from primary resection using an immune checkpoint protein panel. Patient clinical data are summarized in Supplementary Table S1. Most immune checkpoints (e.g., PD-1, PD-L1, LAG3, TIM3) and co-stimulatory molecules (e.g. CD40, ICOS, CD80, CD86) were not expressed at higher in PDAC compared to normal adjacent pancreas (Fig. 1A). However, we found GITR to be the most differentially expressed of all analyzed proteins (Fig. 1A, B). Our findings were confirmed in a larger cohort using the PAAD (PDAC) dataset from the TCGA database, where *GITR* was significantly higher expressed in PDAC compared to normal adjacent pancreas (Fig. 1C).

Next, we decided to investigate the activation of GITR using an agonistic αGITR mAb (DTA-1) in a PDAC mouse model (Pan02), which mimics important aspects observed in human PDAC, such as resistance to therapies, infiltration of immunosuppressive cells such as MDSCs [47] and Treg cells [48] and suppression of effector cells (Supplementary Fig. S1) [49]. Mice were treated twice with agonistic αGITR mAb and tumors were harvested and analyzed on day 14 (Fig. 1D). Tumor growth was monitored throughout the experiment and was significantly reduced by 32% by agonistic GITR treatment compared to control mice that received PBS (Fig. 1E). Next, we tested if this reduced tumor growth would prolong survival of tumor-bearing mice using the same treatment schedule as described above but extending it to 35 days (Fig. 1D). Mice treated with αGITR mAb showed significantly extended survival compared to control mice that received PBS. While no mice in the control group were alive beyond day 18, about 70% of the αGITR mAb-treated mice were still alive on day 18, and 20% of the treated mice had not yet reached the primary endpoint on day 35 (Fig. 1F).

### GITR activation reduces Treg cell number and increases the number of tumor-infiltrating effector cells

We next investigated the cellular effects of GITR in PDAC, by performing flow cytometric analysis of digested tumors of the PBS-treated controls and αGITR-treated mice (Fig. 1G–M, S). Unlike the high infiltration of Treg cells present in PDAC TME of PBS-treated mice, we observed that αGITR mAb treatment reduced CD4^+^ T cell tumor infiltration (Fig. 1G), mainly due to the significant decrease of tumor-infiltrating FoxP3^+^ Tregs (Fig. 1H) compared to control mice that received PBS. Indeed, the number of FoxP3^−^CD4^+^ T cells were similar between αGITR and PBS-treated tumors (Fig. 1I). Furthermore, we found that tumors treated with αGITR mAb showed increased infiltration of NKT cells (Fig. 1J), NK cells (Fig. 1K) and CD80^+^ macrophages (Fig. 1L). These CD80^+^ M1-like macrophages were defined as CD45^+^MHCII^+^CD11b^+^F4/80^+^CD80^+^ and not only the number of CD80^+^ macrophages, but also the expression level of CD80 on macrophages increased, as shown by the mean fluorescence intensity (MFI) (Fig. 1M).

To understand how these specific cells are recruited and/or activated by αGITR mAb treatment, we analyzed the cytokine/chemokine profile within the tumors (Fig. 1N–R). The cytokine IL12p40, which directs proliferation and enhances the cytolytic activity of activated NK cells, was significantly increased after αGITR mAb treatment compared to control mice that received PBS (Fig. 1N), and the same was found for macrophage-colony stimulating factor (M-CSF) (Fig. 1O). The growth factor M-CSF is involved in the proliferation, differentiation and survival of monocytes and macrophages, and in stimulating their phagocytic and chemotactic activity. In addition, macrophage inflammatory protein 1α (MIP-1α), 1β (MIP-1β) and 2 (MIP-2) were also increased after αGITR mAb treatment (Fig. 1P–R). The MIP proteins are chemokines involved in the recruitment of inflammatory cells, such as monocytes/macrophages and neutrophils. As we did not detect significant expression of GITR on these pro-inflammatory, M1-like macrophages, this effect is thought to be indirect, mainly due to the increased expression of M-CSF and the MIP proteins. Altogether, the increase in IL12p40 can lead to improved NK cell function, while the increase in M-CSF and the MIP chemokines plays a role in the recruitment and the increased phagocytic and cytotoxic activity of macrophages in the TME after αGITR mAb treatment. These results are in line with the current literature where GITR activation has been shown to inhibit the suppressor function of Tregs in cancer [50]. Our data show that the main effector cells induced by αGITR mAb treatment are NK and NKT cells, and M1-like macrophages. This data is further supported by flow cytometric analysis of untreated tumors, showing the highest expression of GITR on NKT cells and lymphocytes (Fig. 1S).

### ScRNA-seq reveals GITR-induced activated and cytotoxic tumor-infiltrating lymphocytes

**Fig. 2:**
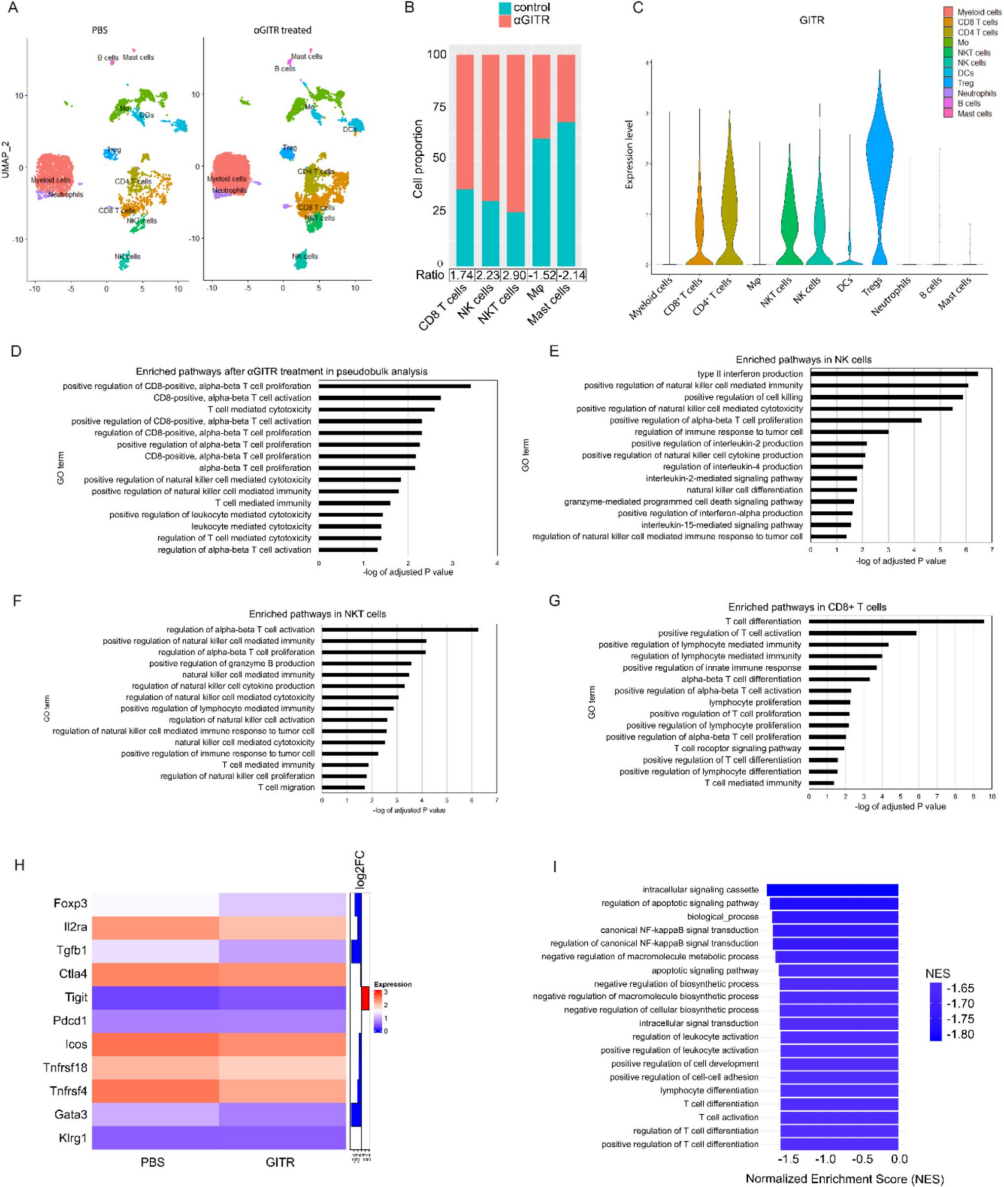
ScRNA sequencing reveals the activated and cytotoxic state of tumor-infiltrating CD8 + T, NK and NKT cells after αGITR mAb treatment. **A** UMAP dimensional reduction. The UMAP is split into αGITR mAb-treated tumors and PBS-treated controls. **B** Cell proportion analysis comparing αGITR mAb-treated tumors and control tumors showing significantly changes cell types. A cutoff for significance was put at a fold change of 1.5. **C** Violin plot showing GITR expression across cell types. **D–G** Significantly enriched pathways after αGITR mAb treatment as calculated by enrichment analysis, (**D**) in pseudobulk analysis, (**E**) in NK cells, (**F**) NKT cells, (**G**) in CD8^+^ T cells. *p* value is corrected for false discovery rate using the Benjamini–Hochberg method. **H** Heatmap showing expression levels and log2FC values of selected genes in PBS and αGITR mAb-treated tumors. **I** Bar plot showing the top 20 downregulated GO terms as calculated by enrichment analysis

To investigate the transcriptomic changes induced by agonistic αGITR mAb treatment, we performed single-cell RNA-sequencing (scRNA-seq) on tumors from 6 treated and 6 untreated mice. Each dataset was integrated using the Harmony package in R. After applying quality control (Supplementary Fig. S2A-C), cell clustering identified 11 clusters, namely NK cells, NKT cells CD4^+^ and CD8^+^ T cells, Treg, DCs, macrophages, neutrophils, B cells, mast cells, and a mixed myeloid cell cluster (Fig. 2A). We first performed cell proportion analysis to visualize the immune cell composition and αGITR mAb treatment induced changes (applied a threshold at a fold change (FC) of + / − 1.5) (Fig. 2B). The most striking changes affect the tumor-infiltrating NK and NKT cells, which present a 2.23 and 2.90-fold increase after αGITR mAb treatment, respectively. These observations are in line with our flow cytometric analysis. In addition, the number of tumor-infiltrating CD8^+^ T cells was increased after αGITR mAb in the TME, whereas the number of macrophages and mast cells was reduced (Fig. 2B). Our scRNA-seq data confirmed the results of the proteomic analysis, showing that *GITR* expression is highest on T, NK, and NKT cells (Fig. 2C).

Next, we performed enrichment analysis using the scRNA-seq dataset as pseudobulk comparing the differentially expressed gene (DEG) profiles of PBS-treated tumors vs αGITR-treated tumors. The overall top enriched pathways upon αGITR mAb treatment were involved in enhanced CD8^+^ T cell proliferation, activation and cytotoxicity, as well as NK cell-mediated immunity and cytotoxicity (Fig. 2D). We next characterized the functional state of NK, NKT, and CD8^+^ T cells by performing pathway enrichment analysis on the individual cell populations (Fig. 2E–G). Specifically, NK cells showed increased cytotoxicity and cytokine production as indicated by pathways such as increased type II interferon production, cell killing and cytotoxicity, and response to tumor cell (Fig. 2E). NKT cell pathways indicated enhanced activation, proliferation, granzyme B production, NK cell cytokine production, cytotoxicity and response to tumor cell (Fig. 2F). Finally, CD8^+^ T cells presented increased proliferation, differentiation, activation, cytotoxicity and TCR signaling pathway (Fig. 2G). Together, these pathway analyses show that NK, NKT and CD8^+^ T cells not only increase in number but are also in an activated and cytotoxic state to eliminate tumor cells.

Additionally, GITR activation was shown to inhibit Treg function and destabilize Treg lineage commitment [18, 50, 51]. Comparing the transcriptional profiles of Treg cells from αGITR-treated with PBS-treated mice, we observed a trend in towards downregulated key immunosuppressive molecules, including Foxp3, Ctla4 and Tgfb1 (Fig. 2H). Gene ontology analysis of the significant Treg DEGs demonstrated an overall suppression of regulatory signaling pathways. Specifically, we observed a strong enrichment in pathways related to T cell differentiation and regulation of apoptosis signaling (Fig. 2I). Thus, these results suggest that αGITR treatment stimulates cytotoxic T and NK cells and inhibits Treg cells.

### PD-L1/PD-1 signaling is reduced, while XCL1/XCR1 signaling is induced upon GITR activation

**Fig. 3:**
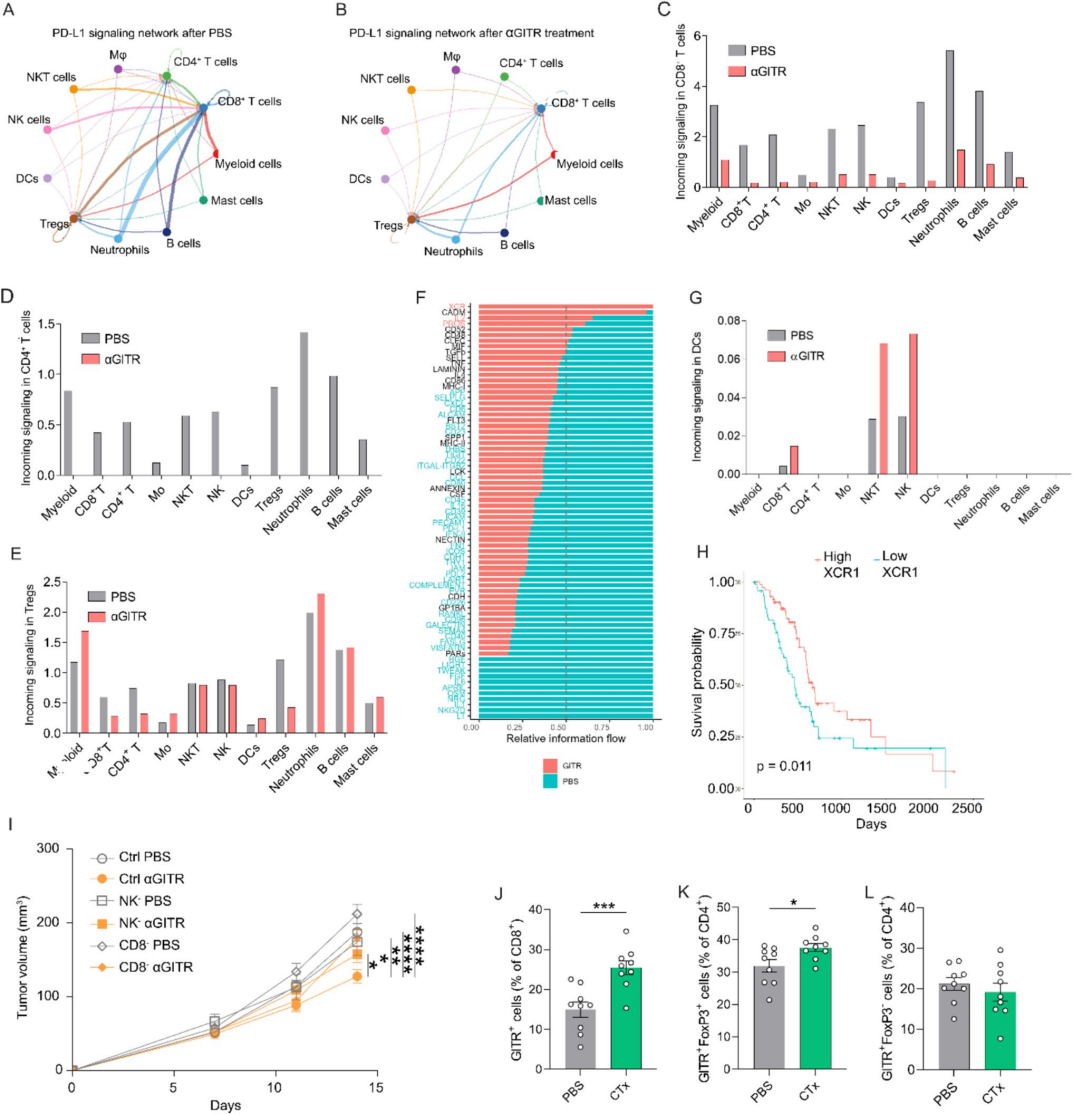
Agonistic GITR treatment reduces PD-L1/PD-1 signaling, while inducing XCL1/XCR1 signaling, and is dependent on NK and CD8^+^ T cells. **A–B** Immune cell communication through PD-L1 (**A**) in PBS-treated and (**B**) αGITR mAb-treated tumors. Line thickness correlates with interaction strength. **C–E** Comparison of the incoming signaling through PD-L1/PD-1 signaling from all immune cells (**C**) in CD8^+^ T cells, (**D**) CD4^+^ T cells and (**E**) Tregs. **F** Bar chart showing relative information flow between cells from PBS-treated and αGITR mAb-treated mice. **G** Comparison of the incoming signaling through XCL1—XCR1 signaling from all immune cells in DCs. **H** Survival plot showing the survival of stage II PDAC patients stratified between high and low XCR1 expression. **I** Tumor growth of mice treated with either αGITR mAb or PBS, together with NK or CD8^+^ T cells depleting antibody or undepleted, n ≥ 12. **J–L** Flow cytometric analysis of isolated tumors stained with anti-GITR antibody, *n* = 9. GITR expression is shown (**J**) on CD8^+^ T cells, (**K**) Treg cells and (**L**) FoxP3^−^ T cells. Error bars show SEM. Statistical significance was calculated using unpaired *t* test; *p* < 0.05*; *p* < 0.01**; *p* < 0.001***; *p* < 0.0001****

To study the cellular interactions and signaling that may induce these functional changes upon αGITR mAb treatment in NK, NKT and CD8^+^ T cells we used the CellChat R package. While in control tumors, most cell types show a strong interaction with CD4^+^ and CD8^+^ T cells through the PD-L1/PD-1 pathway (Fig. 3A), these interactions are completely abrogated in CD4^+^ T cells and strongly reduced in CD8^+^ T cells after GITR activation (Fig. 3B). Quantification of the incoming signaling through PD-L1/PD-1 after GITR treatment showed a strong reduction from all cell types toward CD8^+^ T cells (Fig. 3C), a complete abrogation toward CD4^+^ T cells (Fig. 3D), and slight increase to Tregs (Fig. 3E). The ability of these T cell subsets to interact via this pathway was confirmed by analyzing the expression of *PD-L1* and *PD-1* in these cell types (Supplementary Fig. S2D and E).

Analysis of the relative information flow between cells from αGITR mAb- vs. PBS-treated mice revealed the XCL1/XCR1 signaling pathway as the unique pathway that was significantly increased with αGITR mAb treatment (Fig. 3F) (Supplementary Fig. S3A). Quantification of the incoming signaling through XCL1/XCR1 after GITR treatment showed a strong increase from CD8^+^ T, NK and NKT cells toward DCs (Fig. 3G). Visualizing the differential number of interactions per cell type between the αGITR mAb-treated compared to PBS-treated mice confirmed the increased interactions from NK cells to DCs and from DCs towards cytotoxic T cells (Supplementary Fig. S3B). We confirmed the ability of these cells to interact via the XCL1/XCR1 pathway by showing that *Xcl1* is expressed in CD8^+^ T, NK, and NKT cells (Supplementary Fig. S3C), while *Xcr1* is expressed by DCs (Supplementary Fig. S3D), enabling interaction through the XCL1/XCR1 pathway. The chemokine XCL1/2 is produced by activated NK, NKT, and CD8^+^ T cells, and interaction with its receptor XCR1 leads to the recruitment of conventional type 1 DCs (cDC1) in tumor tissue, which may promote cytotoxic T lymphocyte response via cross-presentation [52]. While XCR1 activation is known to enhance cross-presentation, its role in promoting cytotoxic T cell responses in PDAC requires further investigation. Additionally, enhanced antigen presentation has been shown to increase anti-tumor response leading to prolonged patient survival [53]. Therefore, we used the publicly available TCGA dataset to assess the correlation of *XCR1* expression with patient survival. To exclude any effect of tumor stage on patient survival, we first stratified patients by tumor stage. We found that XCR1 expression does not correlate with patient survival in patients with stage I PDAC (data not shown). However, in patients with stage II PDAC, we found a significant correlation between high XCR1 expression and long-term survival (Fig. 3H). Too few patients presented with stage III and IV tumors to perform survival analysis. To summarize, our data showed that agonistic anti-GITR treatment induced profound changes in the PDAC TME, more specifically: reduced Treg function and PD-1-mediated inhibition of CD8^+^ T cells, while promoting cDC1 activation by cytotoxic T lymphocytes, NK and NKT cells.

### GITR-induced immune responses depend on NK and CD8^+^ T cells

We further investigated the role of NK, NKT, and CD8^+^ T cells in mediating the effect of αGITR mAb treatment by concomitant depletion of these lymphocyte populations. We used the same treatment schedule as previously (Fig. 1D), except that mice were additionally treated with either NK/NKT cell or CD8^+^ T cell depletion Ab, or control Ab. The depletion was complete, as shown by the analysis of the TME on the day of tumor isolation (Supplementary Fig. S3E and G) compared to tumors from undepleted mice (Supplementary Fig. S3F and H). As a result, GITR-induced tumor control was completely abolished in mice depleted of NK/NKT or CD8^+^ T cells compared to undepleted mice receiving αGITR mAb (Fig. 3I). These results show that NK/NKT and CD8^+^ T cells are key effector cells downstream of αGITR mAb treatment and are both required for the treatment to be effective.

### CTx induces GITR expression in CD8^+^ T cells and Tregs in mouse Pan02 tumors

We next investigated whether the current standard neoadjuvant CTx, FOLFORINOX affects GITR expression in mice. For the mouse Pan02 model the same treatment schedule was used as before (Fig. 1D), except that the mice received CTx intra-tumorally (i.t.) on days 7 and 11. At the endpoint, tumors were isolated and processed for flow cytometric analysis (Fig. 3J–L). These experiments showed that compared to control mice that received PBS, CTx treatment increased the number of GITR-expressing cells in CD8^+^ T cells (Fig. 3J) and CD4^+^ Tregs (Fig. 3K), but not in FoxP3^−^CD4^+^ T cells (Fig. 3L). Additionally, the proportion of CD8^+^ T, NK, and NKT cells was significantly increased by the administration of CTx (Supplementary Fig. S3I–K), indicating that CTx treatment promoted a shift towards more pro-inflammatory TME in mice. This data prompted us to investigate whether CTx treatment induces GITR expression in human PDAC patients.

### Neoadjuvant CTx induces GITR expression in human PDAC

**Fig. 4:**
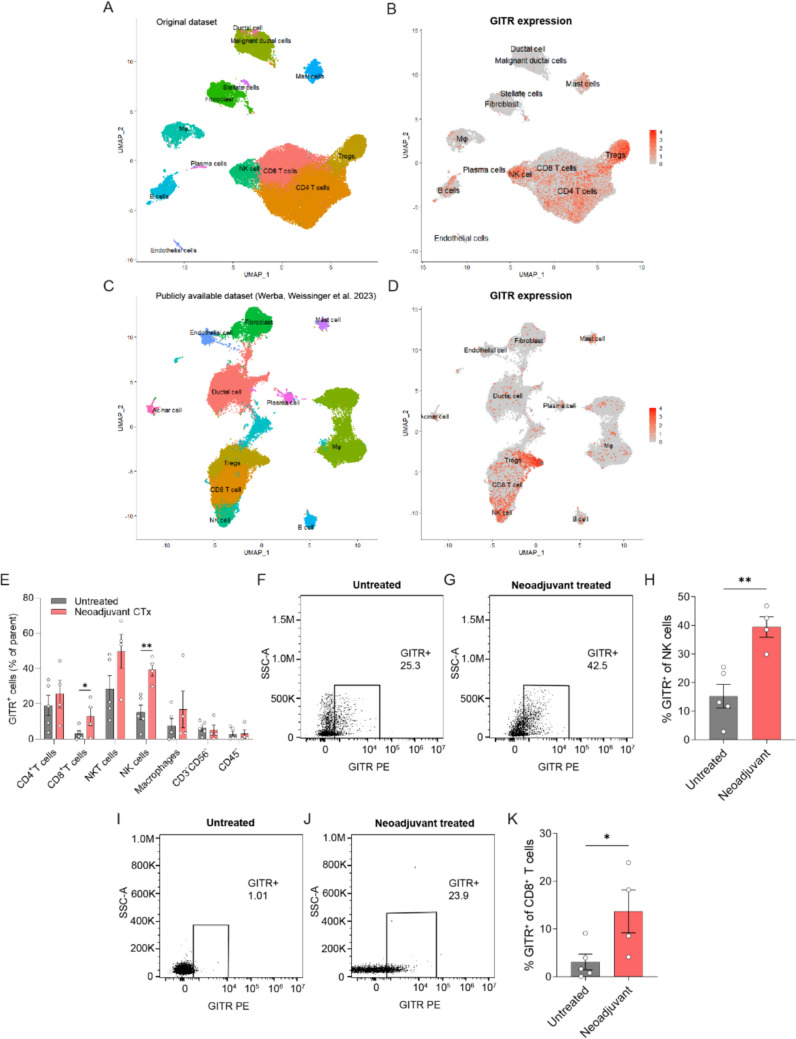
Neoadjuvant therapy induces GITR expression in CD8^+^ T cells and NK cells in human PDAC. Analysis of scRNA seq performed on samples from patients after upfront surgery and neoadjuvant therapy showing (**A**) UMAP dimensional reduction and (**B**) GITR expression overlaid on UMAP. Analysis of scRNA seq data from Werba, Weissinger et al. [31] showing (**C**) UMAP dimensional reduction and (**D**) GITR expression overlaid on UMAP. **E**–**K** Flow cytometric analysis of human tumors stained with anti-GITR antibody. **E** GITR^+^ cells of relevant immune cells. **F**–**G** Representative gating for GITR + NK cells in (**F**) untreated and (**G**) neoadjuvant-treated patients. **H** Percentage GITR^+^ NK cells in untreated and neoadjuvant-treated patients, n ≥ 4. **I**–**J** Representative gating for GITR^+^ CD8^+^ T cells in (**I**) untreated and (**J**) in neoadjuvant-treated patients. **K** Percentage GITR^+^ CD8^+^ T cells in untreated and neoadjuvant-treated patients, n ≥ 4. Statistical significance was calculated using unpaired *t* test; *p* < 0.05*; *p* < 0.01**

To test whether our findings in the Pan02 mouse model are transferable to humans, we compared GITR expression in human PDAC tissue with or without neoadjuvant CTx treatment. Using scRNA-seq, we first analyzed seven PDAC samples from patients who underwent upfront surgery and 6 samples from PDAC patients after neoadjuvant CTx. All patients who received neoadjuvant CTx were treated with FOLFIRINOX. Patient clinical data are summarized in Supplementary Table S2. In brief, patients were selected to ensure equal representation of males and females and to achieve a comparable age distribution. No additional selection criteria were applied, except that patients in the neoadjuvant group were required to demonstrate treatment responsiveness, as assessed by measuring tumor shrinkage by CT scan. Each dataset was integrated using the Harmony package in R. After quality control, cell clustering revealed 12 clusters that were identified as ductal and malignant ductal cells, mast cells, stellate cells, fibroblasts, macrophages, B cells and plasma cells, NK cells, CD4^+^ and CD8^+^ T cells, and Tregs (Fig. 4A). The expression of *GITR* was overlaid on the cell clusters and showed that *GITR* is mainly expressed on Tregs, NK cells and to some extent on CD8^+^ and CD4^+^ T cells (Fig. 4B), which corresponds to our findings in mice and is consistent with the literature [19]. To confirm our findings, we analyzed the published scRNA seq data from Werba, Weissinger et al. [31], which we filtered for primary tumor samples only, resulting in a dataset of 11 samples from untreated patients and 6 from patients who received FOLFORINOX CTx. Each dataset was integrated using the Harmony package in R. Cell clustering revealed 11 different clusters that were identified as fibroblasts, endothelial cells, ductal cells, mast cells, acinar cells, plasma cells, macrophages, Tregs, CD8^+^ T cells, NK cells and B cells (Fig. 4C). Analysis of *GITR* expression showed that GITR is primarily expressed in Tregs, NK, and CD8^+^ T cells (Fig. 4D), in line with our observations in the mouse and human scRNA-seq dataset.

To test whether neoadjuvant CTx also increases GITR protein expression in humans, we processed samples from five patients who had upfront surgery and 4 patients that received neoadjuvant CTx for flow cytometric analysis. Patient clinical data are summarized in Supplementary Table S3 and a representative example of our gating strategy is shown in Supplementary Fig. S4. Similar to our previous observations in our mouse model, most of the GITR expression in human PDAC without neoadjuvant CTx is found in NK, NKT, and CD4^+^ T cells and to a lesser extent in CD8^+^ T cells (Fig. 4E). By comparing samples after neoadjuvant CTx and upfront surgery, we found that neoadjuvant CTx samples showed significantly higher GITR expression in NK cells by a factor of 2.6 (Fig. 4F–H) and in CD8^+^ T cells by a factor of 4.3 (Fig. 4I–K). In addition, the number of T cells was comparable between samples of patients with and without neoadjuvant CTx (Supplementary Fig. S4N), demonstrating that specifically GITR^+^ CD8^+^ T cells were increased by neoadjuvant CTx. While neoadjuvant CTx increases GITR expression in both human and mouse PDAC, there are some differences as GITR is induced in Treg and CD8^+^ T cells in mice, whereas in humans it is induced in NK cells and CD8^+^ T cells. However, the induction of GITR expression in both cases occurred in important effector cells responsible for the response to αGITR mAb treatment. These data suggest that a combination of standard CTx, (that induces GITR expression) and αGITR mAb (that activates the receptor) may be a potential strategy to enhance anti-tumor immunity.

**Fig. 5:**
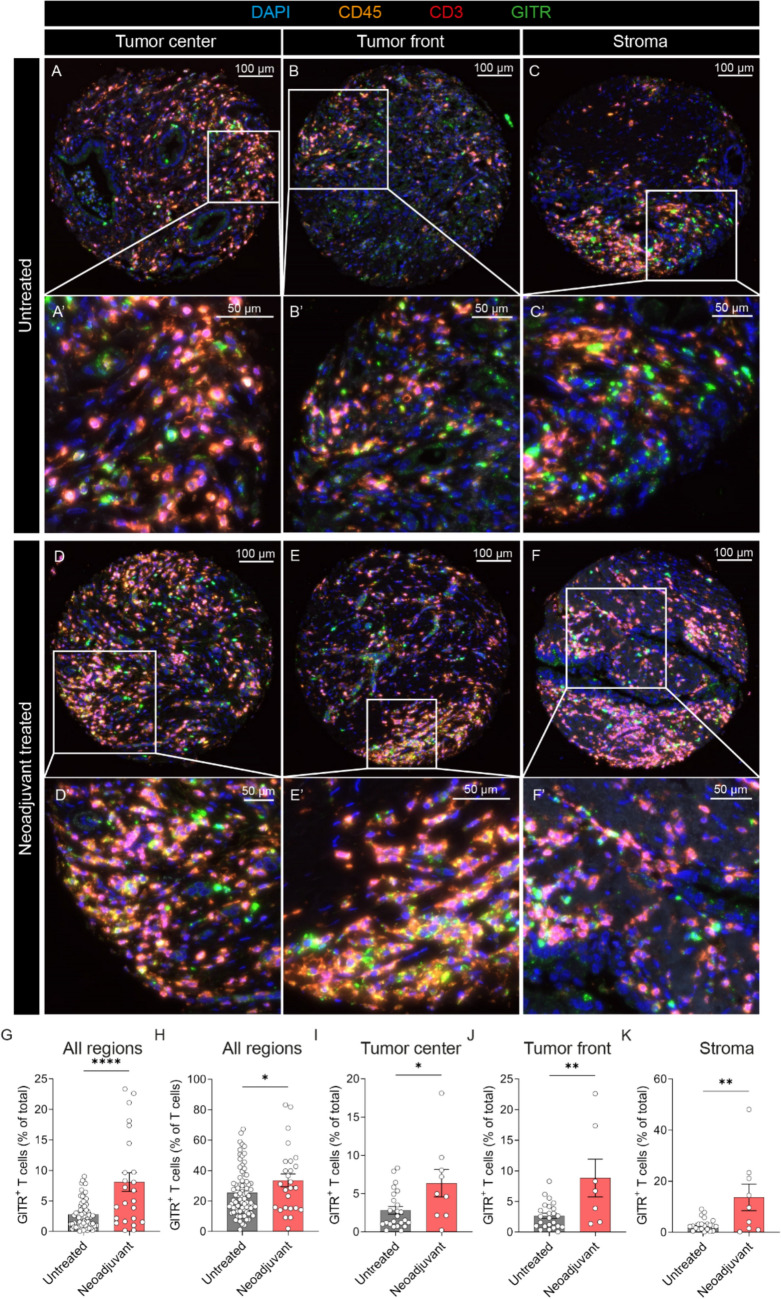
Neoadjuvant therapy increases the number of GITR-expressing T lymphocytes in the tumor microenvironment. Immunofluorescent analysis of a TMA composed of PDAC samples from untreated and neoadjuvant-treated patients. Representative immunofluorescent images of TMA cores **A**–**C’** from untreated patients and **D**–**F’** from neoadjuvant-treated patients. Images from the (**A**, **A’**, **D**, **D’**) tumor center, (**B**, **B’**, **E**, **E’**) tumor front and (**C**, **C’**, **F**, **F’**) stroma are shown. The images (**A’**–**F’**) are numerical magnifications of their respective cores. Percentage of GITR^+^ T cells (**G**) of total cells in all tumor regions, (**H**) of T cells in all tumor regions, (**I**) of total in the tumor center, (**J**) of total at the tumor front, (**K**) of total within the stroma, n ≥ 9. DAPI is shown in blue, CD45 in orange, CD3 in red and GITR in green. Statistical significance was calculated using unpaired *t* test; *p* < 0.05*; *p* < 0.01**; *p* < 0.0001****

To investigate the frequency and spatial distribution of GITR protein-expressing cells, we performed immunofluorescent (IF) imaging of a tissue microarray (TMA) containing 3 cores from 117 patients namely, tumor center, tumor front, and stroma. The cores were filtered based on treatment regimen and 106 cores were retained, 26 from 9 patients who received neoadjuvant CTx and 80 from 28 patients after upfront surgery. Patient clinical data are summarized in Supplementary Table S4. Representative IF images of the TMA cores from untreated patients (Fig. 5A–C’) and neoadjuvant treated patients (Fig. 5D–F’) from each region, tumor center (Fig. 5A, A’, D, D’), tumor front (Fig. 5B, B’, E, E’) and stroma (Fig. 5C, C’, F, F’) are shown. We observed a significant increase in GITR^+^ T cells as a percentage of total cells (Fig. 5G) and as a percentage of total T cells (Fig. 5H) in the tissues from patients who received neoadjuvant therapy. Surprisingly, neoadjuvant therapy significantly increased the infiltration of GITR^+^ T cells in the tumor center (Fig. 5I), the tumor front (Fig. 5J), and in the stroma (Fig. 5K), indicating that GITR expression is increased by neoadjuvant CTx in immune cells, primarily on T lymphocytes.

### In human PDAC, *GITR* expression is spatially restricted to key lymphocyte populations in proximity to the tumor

**Fig. 6:**
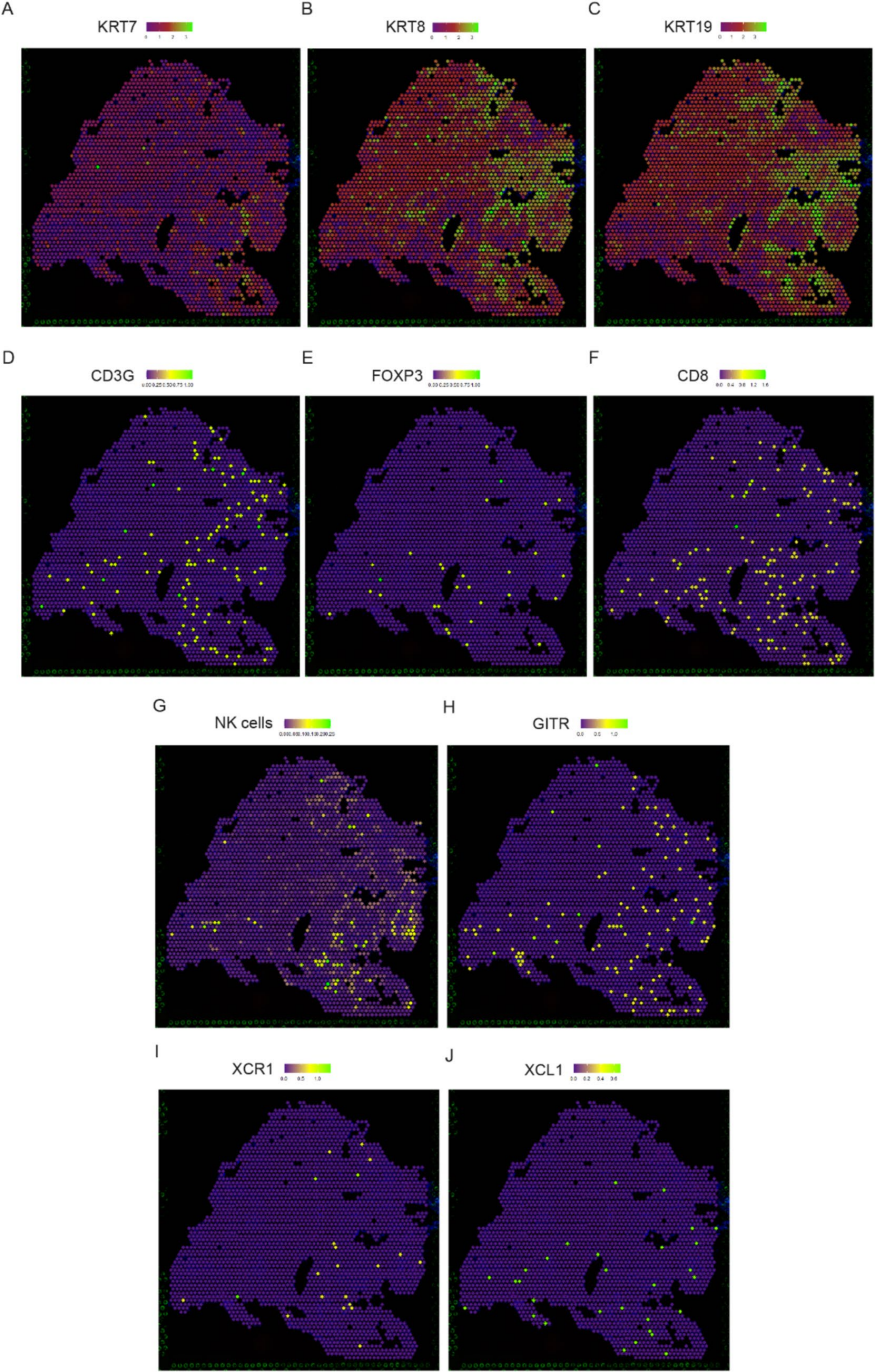
GITR expression in human PDAC is spatially restricted to cells proximal to the tumor, such as Treg, CD8^+^ T and NK cells. Spatial transcriptomics analysis of tissue sections from a representative PDAC patient after neoadjuvant therapy showing the expression of (**A**) KRT7, (**B**) KRT8, (**C**) KRT19, the location of (**D**) CD3G^+^ T lymphocytes, (**E**) FOXP3^+^ Tregs, (**F**) CD8.^+^ T cells, (**G**) NK cells and (**H**) the expression of GITR, (**I**) XCR1 and (**J**) XCL1

Since our scRNA-seq data were derived from dissociated tissue, spatial relationships were not obtained. Therefore, we performed spatial transcriptomic analysis on human samples collected from PDAC patients. In total, we analyzed 4 samples from patients who received neoadjuvant CTx prior to surgery. Patient clinical data are summarized in Supplementary Table S5. Data from one representative patient is shown to allow better comparison across images. First, keratin genes were used to locate the tumor regions in the tissue, specifically *KRT7* (Fig. 6A), *KRT8* (Fig. 6B), and *KRT19* (Fig. 6C). In this representative sample, the tumor areas were mainly located in 3 distinct regions on the right side of the tissue in the picture. Then, by overlaying the expression of *CD3G* (Fig. 6D), *FOXP3* (Fig. 6E) and *CD8* (Fig. 6F) on the tissue section, we identified the location of T lymphocytes, *FOXP3*^+^ Tregs and *CD8*^+^ T cells, respectively, which were present in the tumor regions and located around the tumor cells. To locate the NK cells, data from corresponding scRNA seq were integrated with the spatial transcriptomic data, allowing us to identify NK cells in close proximity to tumor cells (Fig. 6G). Visualizing *GITR* expression shows that expression is primarily located in T cell and NK cell areas (Fig. 6H). Finally, we analyzed the expression of *XCR1* (Fig. 6I) and *XCL1* (Fig. 6J), which were located in the same areas as the *GITR*-expressing cells. Thus, *GITR* expression is colocalized with T cells and NK cells and is in proximity to the tumor to exert its anti-tumor function.

### Higher infiltration of GITR^+^ T lymphocytes in long-term survivors

**Fig. 7:**
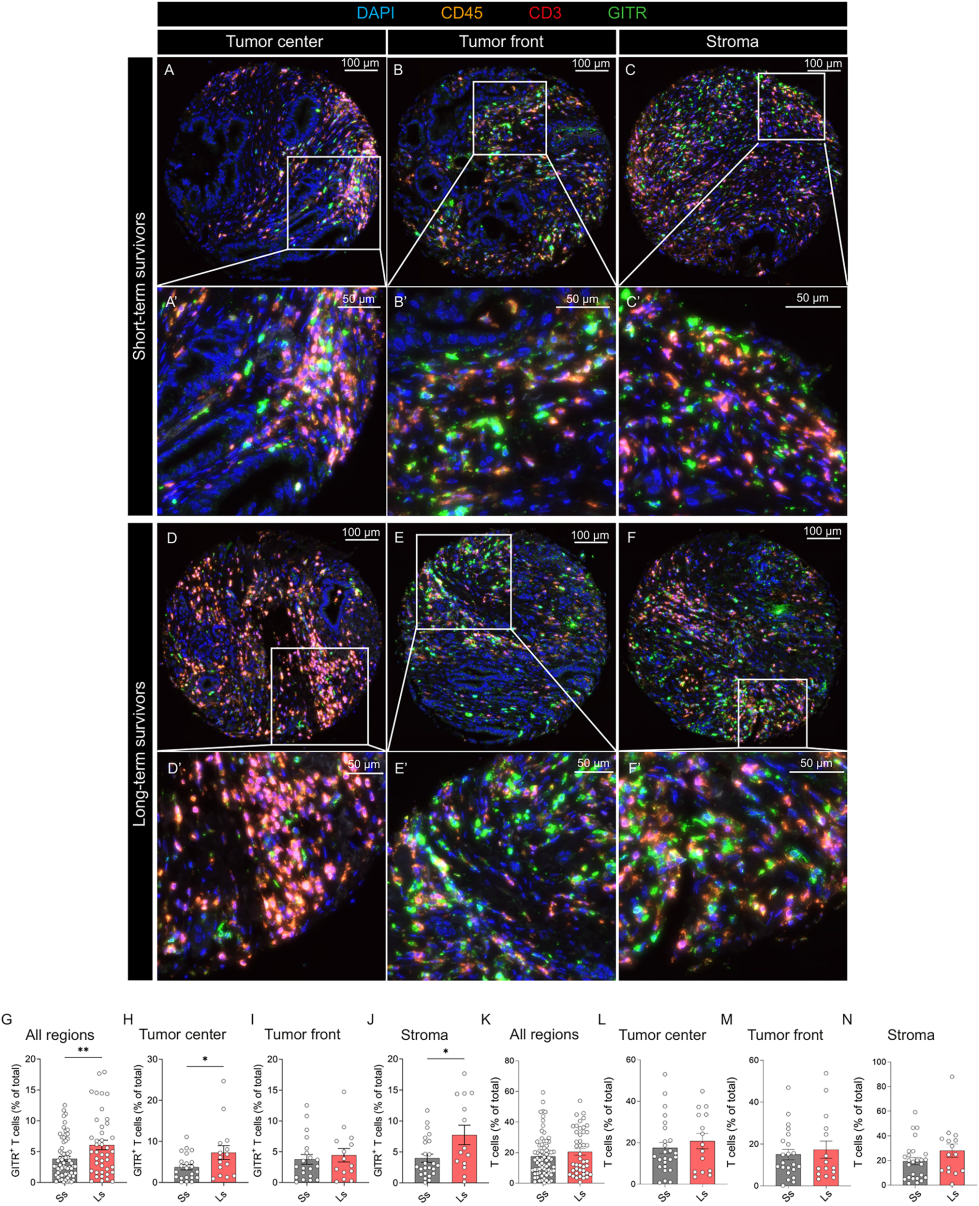
Higher infiltration of GITR^+^ T lymphocytes in long-term survivors. Representative immunofluorescent images of TMA cores from long-term and short-term survivor PDAC patients. **A**–**C’** from short-term survivor patients and (**D**–**F’**) from long-term survivor patients. Images (**A**, **A’**, **D**, **D’**) from the tumor center, (**B**, **B’**, **E**, **E’**) tumor front and (**C**, **C’**, **F**, **F’**) stroma. The images (**A’**–**F’**) are numerical magnifications of their respective cores. Percentage of GITR^+^ T cells (**G**) of total cells irrespective of the tumor region, (**H**) in the tumor center, (**I**) at the tumor front, (**J**) within the stroma, n ≥ 15. T cell percentage (**K**) of total cells irrespective of the tumor region, (**L**) in the tumor center, (**M**) at the tumor front, (**N**) within the stroma, n ≥ 15. DAPI is shown in blue, CD45 in orange, CD3 in red and GITR in green. Statistical significance was calculated using unpaired *t* test; *p* < 0.05*; *p* < 0.01**

We next analyzed whether GITR expression in lymphocytes of the various tumor regions differed between short and long-term survivors. We stratified the TMA cores into 2 groups, the long-term survivors, i.e. patients who survived more than 3 years (45 cores from 17 patients), and the short-term survivors, i.e. patients who survived less than 1 year (74 cores from 22 patients). Patient clinical data are summarized in Supplementary Table S6. Representative IF images of the TMA cores from short-term survivors (Supplementary Fig. 7A–C’) and long-term survivors (Fig. 7D–F’), and from each region, tumor center (Fig. 7A, A’, D, D’), tumor front (Fig. 7B, B’, E, E’), and stroma (Fig. 7C, C’, F, F’) are shown. Quantification showed a significantly higher number of GITR^+^ T lymphocytes in long-term vs. short-term survivors across tumor regions (Fig. 7G). Analyzed per region, we observed a significantly higher number of GITR^+^ T lymphocytes in the tumor center (Fig. 7H), and the stroma (Fig. 7J) but not in the tumor front (Fig. 7I) of long-term survivor patients. To exclude that our findings were due to an overall higher number of lymphocytes in the TME of long-term survivors, we also quantified the number of lymphocytes in each region of the tumor (Fig. 7K–N). The number of lymphocytes was comparable between long-term and short-term survivors in each region of the tumor, indicating that the increased number of GITR^+^ T lymphocyte in long-term survivors is not just a consequence of overall higher lymphocyte counts. To assess the prognostic value of GITR^+^ T lymphocytes and other clinical parameters (including age, gender, extracapsular lymph node (LN), metastasis, and TNM stage), we evaluated their association with survival outcomes (short vs. long survivors), using logistic regression analysis. Three separate logistic regression models were fitted for each tumor sample location (center, front, stroma) and goodness-of-fit was assessed (Hosmer–Lemeshow test: all *p* > 0.05). In all 3 locations, the presence of extracapsular LN metastasis emerged as a significant predictor for short-term survival, whereas the other parameters showed non-significant results (Supplementary Fig. S5). Thus, our analyses revealed higher numbers of GITR^+^ T lymphocytes in long-term survivors, suggesting a potential influence on patient survival outcomes. However, in this study, extracapsular lymph-node metastasis was the only independent predictor of short-term survival, potentially obscuring the effects of other variables.

## Discussion

PDAC is one of the most aggressive cancers, with a low survival rate in diagnosed patients. In addition, standard treatment options are limited to surgical resection and/or CTx. Immunotherapy, although being successful in various cancers, has been poorly effective in treating pancreatic cancer. In this study, we showed that GITR expression is upregulated in PDAC, both intrinsically and in response to chemotherapy (FOLFIRINOX). Importantly, GITR upregulation does not necessarily imply its functional activation, as ligand availability and receptor engagement are required to initiate downstream signaling, but suggests that these tumors may be more responsive to subsequent GITR-targeted activation. Additionally, we explored the in vivo effects of GITR activation (αGITR mAb) and demonstrated that GITR activation reshapes the PDAC TME toward a more immunogenic and tumor suppressive state. Thus, we hypothesize that GITR-targeted therapy could enhance the efficacy of standard CTx through additive or even synergistic effects on the anti-tumor immune response. Based on these findings, we present GITR-targeted therapy as a novel anti-tumor and immunomodulatory treatment option and as a potentially valuable adjunct to standard neoadjuvant CTx in the treatment of PDAC. Further preclinical studies are warranted to explore the anti-tumor efficacy of such combination therapy.

Most PDAC patients do not present symptoms until advanced stages, leading to a delayed diagnosis, which can extend over several months [54]. Therefore, new therapies are needed to treat tumors at an advanced stage. Here, we show in preclinical studies that targeting GITR suppressed established tumors and prolonged survival in the PDAC mouse model, as we started αGITR mAb treatment when the tumor was already established.

By performing cellular and molecular analysis, we found that αGITR mAb treatment reduced the number and activity of Tregs. It has been demonstrated that high infiltration of Tregs in the TME correlates with increased tumor volume and poor prognosis in PDAC patients [55]. Similar results were obtained using αGITR therapy in preclinical models of glioblastoma, where agonistic αGITR antibody promoted Treg differentiation into effector CD4^+^ T cells, reduced Treg-specific inhibition of the anti-tumor response and induced anti-tumor effector cells. However, at least parts of the effects of αGITR-targeted therapy seem to be specific to the cancer type, as the number of Tregs in the TME was not affected by the treatment in the glioblastoma mouse model [56]. In addition, in PDAC we found that αGITR mAb treatment increased the number of effector CD8^+^ T, NK, and NKT cells, as well as M1-like macrophages in the TME. It has been shown that the absolute number of NK cells prior to therapy correlates positively with PDAC patient survival [57]. Furthermore, the loss of NKT cells in the LSL-Kras^G12D/+^ mouse model was shown to increase PanIN lesions and M2 macrophage infiltration [58]. Furthermore, the NKT cell infiltration rate positively correlates with a favorable prognosis in PDAC patients [59]. Lastly, the increase of M1-like macrophages in the TME holds great promise for the treatment of PDAC as the use of anti-CD40 treatment recruits and activates tumoricidal M1-like macrophages, which facilitated the depletion of tumor stroma and subsequently increased CTx response [60]. The observed discrepancy between the cellular proportions of the flow cytometry and scRNAseq results can be explained by the non-linear correlation between proteomic measurements and transcriptomic readouts [61, 62].

The GITR signaling pathway has been shown to be TRAF2/5-dependent and to induce NFκB, which is associated with Bcl-xL upregulation and IL-9 induction, suggesting a potential role of GITR in enhancing T cell survival [63, 64], as well as improved function of DCs and cytotoxic T cells [65]. In addition, GITR signaling has been shown to lower the threshold for CD28 co-stimulation in CD8^+^ T cells [66], induce the expression of 4-1BB, and promote the survival of CD8^+^ memory T cells [67, 68]. Here we show that the recruitment and activation of effector CD8^+^ T,NK and NKT cells, as well as M1-like macrophages may occur through IL12p40, M-CSF, MIP-1α, MIP-1β and MIP-2 signaling, as their concentration was significantly higher in the TME of αGITR mAb-treated mice. In vitro experiments have shown that pancreatic cancer cells reduce IL12p40 production [69], thereby reducing NK cell activation and cytotoxicity. Here we show that αGITR mAb treatment can restore the production of IL12p40. In addition, the serum concentration of MIP-1β in human patients has recently been shown to be a biomarker of response to FOLFIRINOX therapy [70].

By performing scRNA-seq, we describe the activated and cytotoxic state of tumor-infiltrating NK, NKT and CD8^+^ T cells in αGITR-treated mice and GITR^+^ cells in PDAC patient samples. In a recent study on gastric and colon cancer patient-derived tumor culture, agonistic αGITR was shown to increase effector gene expression in cytotoxic CD8^+^ T cells, but not induce any transcriptomic response in exhausted CD8^+^ T cells [71]. In our study, we show that the changes induced by agonistic αGITR therapy in exhausted CD8^+^ T cells occur at the signaling level instead of the transcriptional level. Indeed, by applying cell–cell communication analysis to our data, we demonstrated the reduced communication between immune cells through PD-1/PD-L1. In addition, αGITR-treatment specifically induced XCL1/XCR1 signaling. Recently, it has been shown in the Pan02 mouse model that activating NK cells by blocking tumor-derived prostaglandin (PGE2) leads to increased XCL1 secretion, which in turn improves the migration of immature DC, enhancing antigen-associated innate immune cell response [72].

Multiple scRNA-seq datasets from human and mouse samples allowed us to demonstrate that NK, NKT and CD8^+^ T cells, as well as Tregs, are the main *GITR*-expressing cells in PDAC. By specifically depleting NK and CD8 + T cells, we confirmed that these are the primary effector cells of GITR activation as their depletion completely abrogated any beneficial effect of αGITR mAb treatment on tumor growth. In addition, we showed that *GITR* expression is spatially restricted to NK, *FOXP3*^+^ Tregs, and CD8^+^ T cells surrounding the tumor regions in human PDAC patients. Furthermore, we showed that the current standard-of-care neoadjuvant CTx induces GITR expression in CD8^+^ T cells and Tregs in an in vivo model, and in CD8^+^ T and NK cells in human patients. It is known that neoadjuvant CTx such as FOLFIRINOX is not only cytotoxic but may facilitate a pro-inflammatory TME by reducing infiltration of Tregs, MDSCs and M2 macrophages, increasing infiltration of tumor-specific effector cells such as CD8^+^ T cells, and decreasing stromal activation [10, 73]. Furthermore, the extent of antitumor immune remodeling correlates with the degree of response to neoadjuvant therapy [11, 74, 75]. Using IF image analysis, we showed that this neoadjuvant-specific GITR-induction occurs in T lymphocytes in the tumor center, front and stroma. Thus, our data suggest that GITR may play a pivotal role in immunomodulation of PDAC TME after neoadjuvant therapy. Indeed, it has been shown that high infiltration of T cells predicts better survival and that infiltration in the tumor center has the greatest impact on patient survival [76].

Finally, we showed that the number of GITR^+^ T lymphocytes was significantly higher in long-term survivors. However, further studies with larger patient cohorts are needed to determine the prognostic significance of the number of GITR^+^ T lymphocytes in PDAC tumors.

By combining mouse models and patient samples, we characterized the αGITR-treatment-induced effects in PDAC. However, we acknowledge that this study has several limitations and that further preclinical testing and validation is needed. First, the number of samples in this study is limited; Second, the patient cohort has selection bias, as only FOLFIRINOX-responding patients would proceed to surgical removal of the tumor; and third each in vivo model has inherent limitations, and existing models insufficiently recapitulate all critical human PDAC features to facilitate successful clinical translation, it is important to evaluate the therapeutic effects of mono and combination therapy across various models and in clinical studies [77].

In conclusion, our findings provide the first preclinical evidence for the therapeutic potential of agonistic anti-GITR treatment in PDAC, suggesting further preclinical investigation in combination with standard-of-care neoadjuvant chemotherapies for PDAC patients.

## Supplementary Information

Below is the link to the electronic supplementary material.

Supplementary file1 (PDF 42476 KB)
