# Supplementary material for: Agonistic GITR treatment enhances antitumor immune responses and suppresses tumor progression in pancreatic ductal adenocarcinoma
Source: J Gastroenterol. 2026 Feb 1;61(5):638–59. doi: 10.1007/s00535-026-02347-y (PMC13157448; doi:10.1007/s00535-026-02347-y)
Supplement: Supplementary file 1 — Supplementary file1 (PDF 42476 KB) [file 535_2026_2347_MOESM1_ESM.pdf]

A Pancreas and PDAC histology

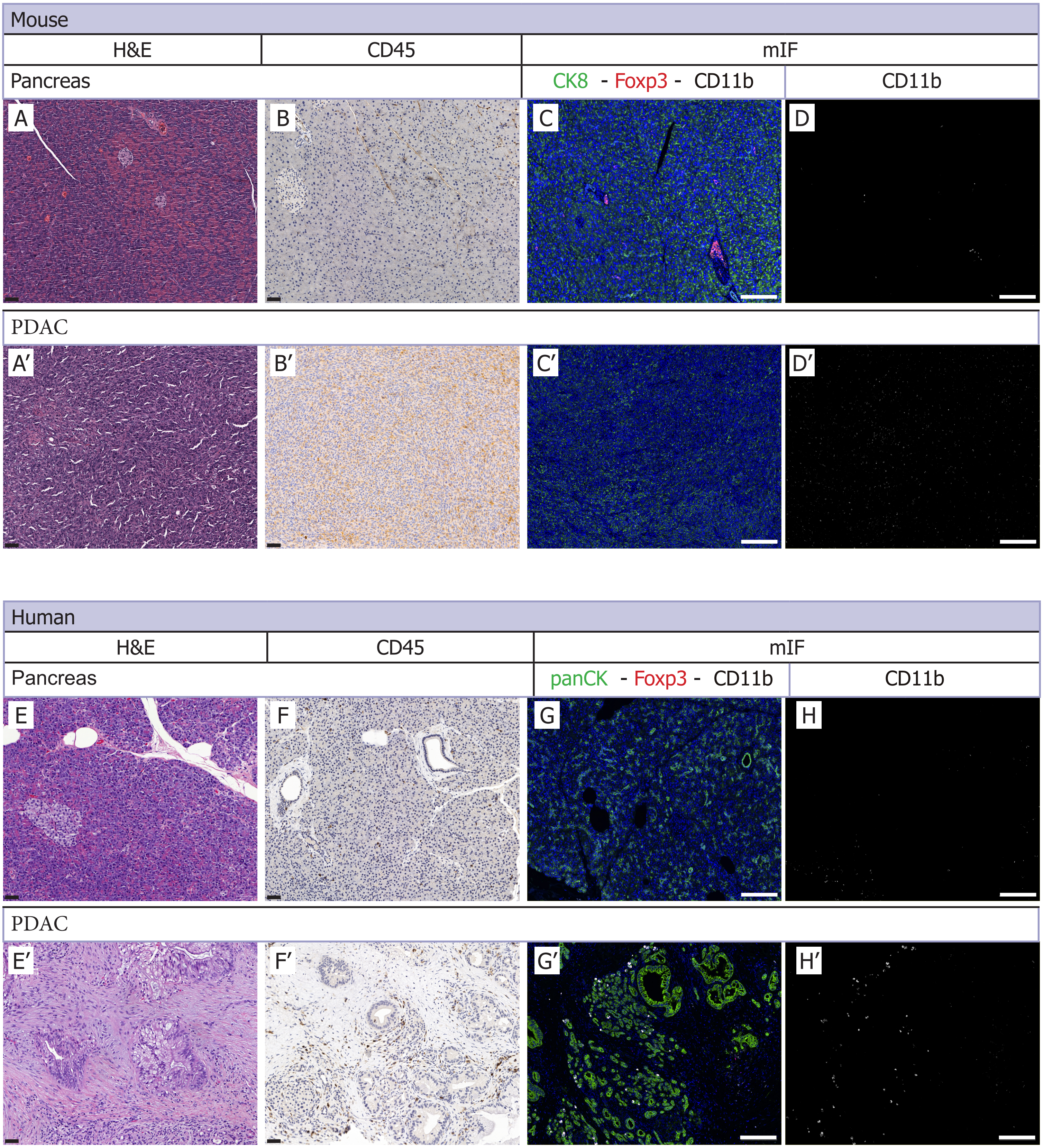

Supplementary Figure S1: Histology of mouse and human pancreas and PDAC tissue. Histological stainings of **A-D)** healthy mouse pancreas tissue, **A',-D')** mouse PDAC tissue, **E-H)** human normal adjacent tissue and **E'-H')** human PDAC tissue. Scale bars are 50µm. **A,A',E,E')** Hematoxylin & Eosin staining. **B,B',F,F')** Immunohistochemistry staining CD45. Scale bars are 50µm. **C,D,C',D',G,H,G',H')** Multiplex immunofluorescent staining (mIF). DAPI is shown in blue, Pan-CK (human)/ CK-8 (mouse) staining acinar, ductal and cancer cells shown in green, FOXP3 staining Treg cells shown in red and CD11b staining myeloid cells shown in grey. Scale bars are 200µm. **D,D',H,H')** Grey scale of CD11b staining. Scale bars are 200 µm.

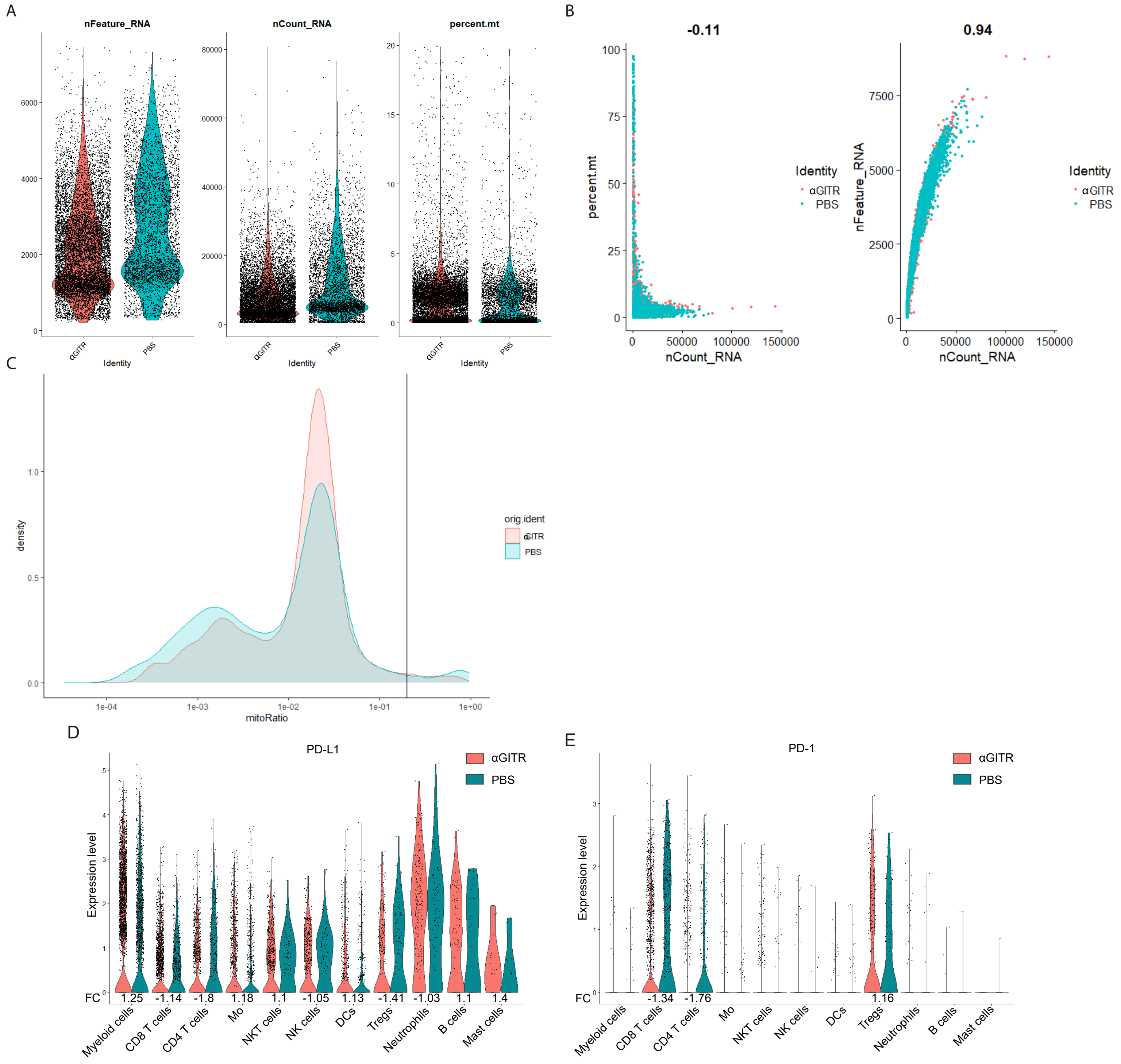
A
nFeature_RNA
nCount_RNA
percent.mt
Identity
αGITR
PBS
B
-0.11
0.94
percent.mt
nCount_RNA
nFeature_RNA
Identity
αGITR
PBS
C
density
mitoRatio
orig.ident
αGITR
PBS
D
PD-L1
Expression level
FC
1.25 -1.14 -1.8 1.18 1.1 -1.05 1.13 -1.41 -1.03 1.1 1.4
Myeloid cells
CD8 T cells
CD4 T cells
Mo
NKT cells
NK cells
DCs
Tregs
Neutrophils
B cells
Mast cells
E
PD-1
Expression level
FC
-1.34 -1.76 1.16

Supplementary Figure S2: Quality control of the scRNA seq data reveals high quality samples, and expression of PD-1/PD-L1 is lower in αGITR treated mice, while XCL1/XCR1 is increased. **A)** Violin plots showing the number of genes detected in each cell (nFeature), the total number of molecules detected within a cell (nCount) and the percentage of mitochondrial RNA (percent.mt). **B)** Scatterplots where nCount is plotted against precent.mt (left) and nFeature (right), to identify low quality cells and doublets. **C)** Density plot showing the density of cells based on their ratio of mitochondrial RNA content. The vertical bar is the threshold used to filter out cells of low quality. **D-E)** Violin plots showing **D)** PD-L1, **E)** PD-1 expression across cell types, split by treatment, with corresponding fold change (FC).

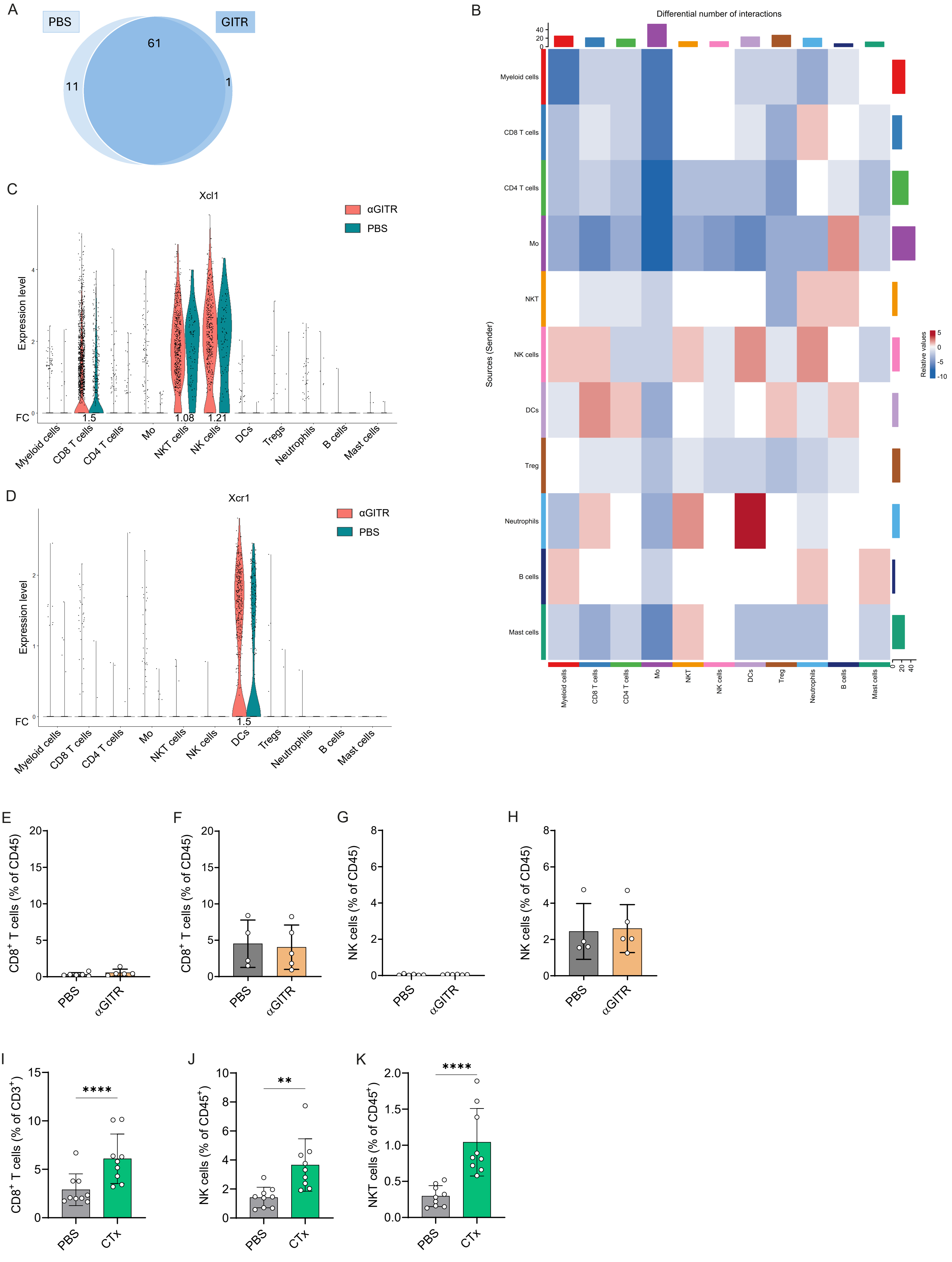
A
PBS
GITR
61
11
1
B
Differential number of interactions
Sources (Sender)
Relative values
Myeloid cells
CD8 T cells
CD4 T cells
Mo
NKT
NK cells
DCs
Treg
Neutrophils
B cells
Mast cells
C
Xcl1
αGITR
PBS
Expression level
FC
1.5
1.08
1.21
Myeloid cells
CD8 T cells
CD4 T cells
Mo
NKT cells
NK cells
DCs
Tregs
Neutrophils
B cells
Mast cells
D
Xcr1
αGITR
PBS
Expression level
FC
1.5
E
CD8+ T cells (% of CD45)
PBS
αGITR
F
CD8+ T cells (% of CD45)
PBS
αGITR
G
NK cells (% of CD45)
PBS
αGITR
H
NK cells (% of CD45)
PBS
αGITR
I
CD8+ T cells (% of CD3+)
****
PBS
CTx
J
NK cells (% of CD45+)
**
PBS
CTx
K
NKT cells (% of CD45+)
****
PBS
CTx

Supplementary Figure S3: scRNA analyses of PBS and αGITR treated tumors, efficient CD8$^+$ T and NK cell depletion and increased number of CD8$^+$ T and NK after CTx. **A)** Venn diagram showing the significant signaling pathways for each condition and the overlap. **B)** Heatmap showing the differential number of interactions between cell types of PBS and αGITR treated mice. **C-D)** Violin plots showing **C)** Xcl1, **D)** Xcr1 expression across cell types, split by treatment, with corresponding fold change (FC). **E-K)** Flow cytometry results. The percentage of **E-F)** CD8$^+$ T cells and **G-H)** NK cells is shown for both αGITR treated and PBS treated tumors after treatment with **E, G)** respective depletion Ab and **F, H)** control Ab, n=6. **I-K)** Flow cytometric analysis of isolated tumors stained with antibodies against **I)** CD8, **J-K)** NK1.1 and **K)** CD3. Statistical significance was calculated using unpaired t-test; $p < 0.01$ ** ; $p < 0.0001$ ****.

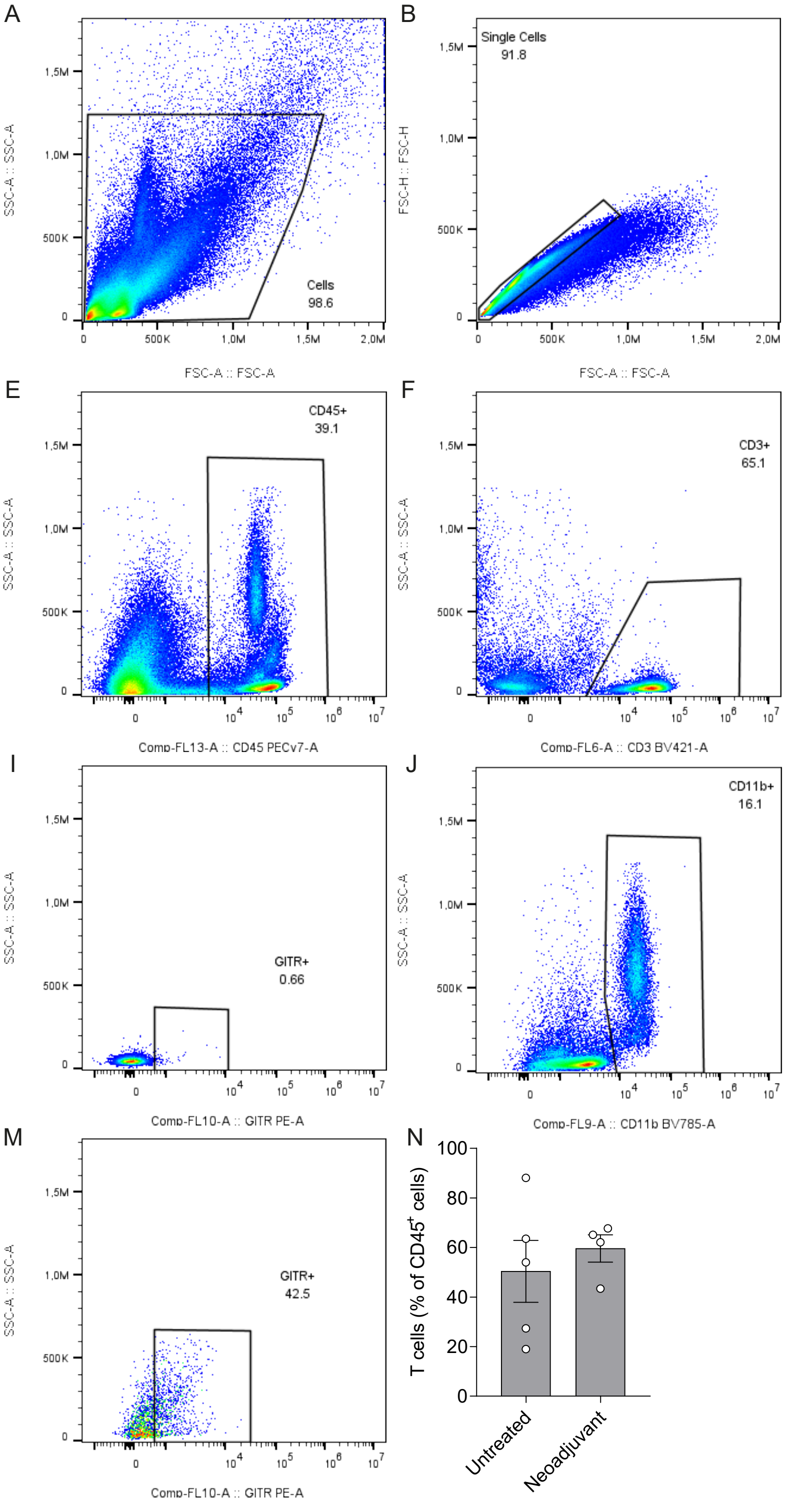

A
Cells
98.6
SSC-A :: SSC-A
FSC-A :: FSC-A
B
Single Cells
91.8
FSC-H :: FSC-H
FSC-A :: FSC-A
C
Live
49.4
SSC-A :: SSC-A
Comp-FL7-A :: Zombie BV510-A
D
Non-epithelial
99.7
Comp-FL9-A :: CD11b BV785-A
Comp-FL1-A :: CD56 Alexa488-A
E
CD45+
39.1
SSC-A :: SSC-A
Comp-FL13-A :: CD45 PECy7-A
F
CD3+
65.1
SSC-A :: SSC-A
Comp-FL6-A :: CD3 BV421-A
G
CD8+_2
27.9
CD4+_2
70.5
Comp-FL3-A :: CD8 APC-A
Comp-FL5-A :: CD4 APC-Cy7-A
H
GITR
7.55
SSC-A :: SSC-A
Comp-FL10-A :: GITR PE-A
I
GITR+
0.66
SSC-A :: SSC-A
Comp-FL10-A :: GITR PE-A
J
CD11b+
16.1
SSC-A :: SSC-A
Comp-FL9-A :: CD11b BV785-A
K
GITR+
3.30
SSC-A :: SSC-A
Comp-FL10-A :: GITR PE-A
L
CD56+
4.05
SSC-A :: SSC-A
Comp-FL1-A :: CD56 Alexa488-A
M
GITR+
42.5
SSC-A :: SSC-A
Comp-FL10-A :: GITR PE-A
N
T cells (% of CD45+ cells)
Untreated
Neoadjuvant

Supplementary Figure S4: Representative gating strategy used to analyze human PDAC samples and T cell proportion. The proportion of gated cells are shown for **A)** cells, **B)** singlets, **C)** living cells, **D)** non-epithelial cells, **E)** $CD45^{+}$ cells, **F)** $CD3^{+}$ cells, **G)** $CD4^{+}$ and $CD8^{+}$ cells, **H)** $GITR^{+}$ of $CD4^{+}$ cells, **I)** $GITR^{+}$ of $CD8^{+}$ cells, **J)** $CD11b^{+}$ cells, **K)** $GITR^{+}$ of $CD11b^{+}$ cells, **L)** $CD56^{+}$ cells and **M)** $GITR^{+}$ of $CD56^{+}$ cells. **N)** T cell proportion in untreated and neoadjuvant treated patient samples, n≥4.

A **Center: Forest plot showing odds ratios with 95% confidence intervals**

Variable	Odds Ratio	95% CI	P-value	Plot
Age	0.99	(0.86 - 1.13)	0.836	
Gender (Male vs Female)	1.47	(0.23 - 11.67)	0.694	
Extracapsular LN metastases (Yes vs No)	92.84	(7.26 - 5923.61)	0.005	
TNM Stage (Early vs Advanced)	1.66	(0.20 - 13.91)	0.631	
% GITR+ T cells / Total cells	1.24	(1.03 - 1.59)	0.040	
% Total T cells / Total cells	0.87	(0.74 - 0.99)	0.055	

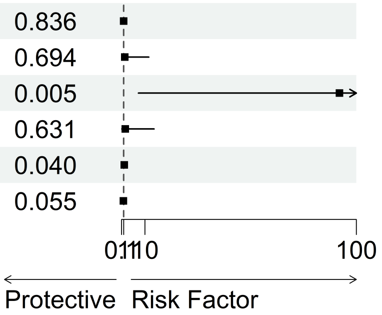

B **Front: Forest plot showing odds ratios with 95% confidence intervals**

Variable	Odds Ratio	95% CI	P-value	Plot
Age	1.00	(0.90 - 1.11)	0.966	
Gender (Male vs Female)	1.50	(0.28 - 9.50)	0.645	
Extracapsular LN metastases (Yes vs No)	41.48	(4.48 - 1323.03)	0.006	
TNM Stage (Early vs Advanced)	2.07	(0.34 - 13.90)	0.435	
% GITR+ T cells / Total cells	0.91	(0.73 - 1.11)	0.401	
% Total T cells / Total cells	1.02	(0.94 - 1.11)	0.639	

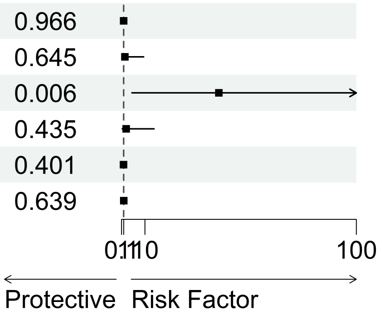

C **Stroma: Forest plot showing odds ratios with 95% confidence intervals**

Variable	Odds Ratio	95% CI	P-value	Plot
Age	0.98	(0.88 - 1.10)	0.737	
Gender (Male vs Female)	3.90	(0.63 - 38.32)	0.177	
Extracapsular LN metastases (Yes vs No)	50.26	(4.91 - 1563.15)	0.005	
TNM Stage (Early vs Advanced)	5.40	(0.79 - 53.19)	0.104	
% GITR+ T cells / Total cells	0.91	(0.71 - 1.05)	0.365	
% Total T cells / Total cells	1.03	(0.95 - 1.14)	0.488	

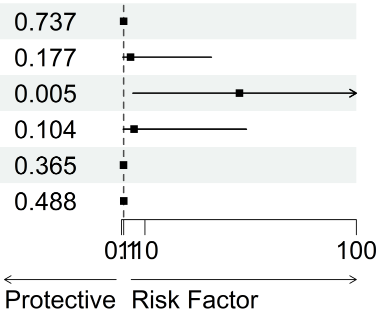

Supplementary Figure S5: Results of multivariate logistic regression analysis for 3 sample locations: **A)** tumor center, **B)** front, and **C)** stroma including the Odds ratio, 95% CI, and p-value for each variable and a summary plot.

	Patients (*n* = 15)
**Age, years, mean (St.Err)**	69 (3.1)
**Sex, *n* (%)**	
Male	7 (46.7)
Female	8 (53.3)
**Relapse, *n* (%)**	
Yes	8 (53.3)
No	7 (46.7)
**PFS if relapse, days, mean (St.Err)**	350 (54.3)
**Deceased, *n* (%)**	
Yes	10 (66.7)
No	5 (33.3)
**OS if deceased, days, mean (St.Err)**	419 (98.9)
**Location of primary tumor, *n* (%)**	
Caput	14 (93.3)
Corpus and cauda	1 (6.7)
**Tumor stage, *n* (%)**	
I	4 (26.7)
II	7 (46.7)
III	4 (26.7)
IV	0 (0)
**Extracapsular extension to LN, *n* (%)**	
Yes	12 (80)
No	3 (20)
**Resection type, *n* (%)**	
Whipple	9 (60)
Total pancreatectomy	6 (40)
**Neoadjuvant CTx, *n* (%)**	
FOLFIRINOX	2 (13.3)
**Comorbidities, *n* (%)**	
Postoperative diabetes	6 (40)
Diabetes mellitus type 2	2 (13.3)
Breast cancer	2 (13.3)
Fibromyalgia	1 (6.7)
Nephrolithiasis	1 (6.7)
Sarcoidosis	1 (6.7)

Supplementary Table S1: Clinical characteristics of patients. Samples from surgical PDAC resection used for the Human Immuno-Oncology Checkpoint 16-plex assay from Eve Technologies.

	Untreated (*n* = 7)	Neoadjuvant (*n* = 6)
**Age, years, mean (St.Err)**	65 (4.2)	59 (6.3)
**Sex, *n* (%)**		
Male	4 (57.1)	3 (50)
Female	3 (42.9)	3 (50)
**Relapse, *n* (%)**		
Yes	4 (57.1)	2 (33.3)
No	3 (42.9)	4 (66.7)
**PFS if relapse, days, mean (St.Err)**	346 (95.1)	253 (154)
**Deceased, *n* (%)**		
Yes	2 (28.6)	1 (16.7)
No	5 (71.4)	5 (83.3)
**OS if deceased, days, mean (St.Err)**	281 (153.5)	295 (0)
**Location of primary tumor, *n* (%)**		
Caput	5 (71.4)	2 (33.3)
Corpus	0 (0)	4 (66.7)
Cauda	1 (14.3)	0 (0)
Ampulla Vateri	1 (14.3)	0 (0)
**Tumor stage, *n* (%)**		
I	0 (0)	1 (16.7)
II	4 (57.1)	3 (50)
III	3 (42.9)	0 (0)
IV	0 (0)	2 (33.3)
**Extracapsular extension to LN, *n* (%)**		
Yes	5 (71.4)	4 (66.7)
No	2 (28.6)	2 (33.3)
**Resection type, *n* (%)**		
Whipple	5 (71.4)	0 (0)
Distal pancreatectomy	1 (14.3)	0 (0)
Total pancreatectomy	1 (14.3)	6 (100)
**Neoadjuvant CTx, *n* (%)**		
FOLFIRINOX	0 (0)	6 (100)
**Neoadjuvant CTx cycles, n, mean (St.Err)**	0 (0)	8.6 (1)
**Comorbidities, *n* (%)**		
Postoperative diabetes	2 (38.6)	6 (100)
Diabetes mellitus type 2	1 (14.3)	1 (16.7)
Renal insufficiency	1 (14.3)	0 (0)
Gastritis	1 (14.3)	0 (0)
Dislipidemy	1 (14.3)	0 (0)

Supplementary Table S2: Clinical characteristics of patients after neoadjuvant chemotherapy or upfront surgery. Samples from surgical PDAC resection used for scRNA sequencing analysis.

	Untreated (*n* = 5)	Neoadjuvant (*n* = 4)
**Age, years, mean (St.Err)**	59 (4.4)	70 (4.3)
**Sex, *n* (%)**		
Male	3 (60)	3 (75)
Female	2 (40)	1 (25)
**Relapse, *n* (%)**		
Yes	0 (0)	0 (0)
No	5 (100)	4 (100)
**PFS if relapse, days, mean (St.Err)**	N/A	N/A
**Deceased, *n* (%)**		
Yes	0 (0)	1 (25)
No	5 (100)	3 (75)
**OS if deceased, days, mean (St.Err)**	N/A	21 (0)
**Location of primary tumor, *n* (%)**		
Caput	0 (0)	3 (75)
Corpus	2 (40)	1 (25)
Cauda	0 (0)	0 (0)
Censored	3 (60)	0 (0)
**Tumor stage, *n* (%)**		
I	0 (0)	0 (0)
II	3 (60)	1 (25)
III	1 (20)	2 (50)
IV	0 (0)	1 (25)
Censored	1 (20)	0 (0)
**Extracapsular extension to LN, *n* (%)**		
Yes	3 (60)	3 (75)
No	1 (20)	1 (25)
Censored	1 (20)	0 (0)
**Resection type, *n* (%)**		
Whipple	1 (20)	2 (50)
Distal pancreatectomy	3 (60)	1 (25)
Total pancreatectomy	1 (20)	1 (25)
**Neoadjuvant CTx, *n* (%)**		
FOLFIRINOX	0 (0)	4 (100)
**Neoadjuvant CTx cycles, n, mean (St.Err)**	N/A	8 (1.8)
**Comorbidities, *n* (%)**		
Postoperative diabetes	1 (20)	1 (25)
Diabetes mellitus type 2	0 (0)	1 (25)
Diabetes mellitus type 3	0 (0)	1 (25)
Renal insufficiency	0 (0)	1 (25)
Hypertensive heart disease	1 (20)	0 (0)

Supplementary Table S3: Clinical characteristics of patients after neoadjuvant chemotherapy or upfront surgery. Samples from surgical PDAC resection used for flow cytometric analysis.

	Untreated (*n* = 28)	Neoadjuvant (*n* = 9)
**Age, years, mean (St.Err)**	70 (1,8)	68 (1.9)
**Sex, *n* (%)**		
Male	17 (60,7)	6 (66.7)
Female	11 (39,3)	3 (33,3)
**PFS, days, mean (St.Err)**	320 (50.7)	701 (195.1)
**OS, days, mean (St.Err)**	261 (58.3)	699 (187.1)
**Precursor lesion, *n* (%)**		
IPMN	2 (7.1)	0 (0)
ADM	1 (3.6)	0 (0)
PanIN low grade	1 (3.6)	0 (0)
PanIN high grade	20 (71.4)	8 (88.9)
NA	4 (14.3)	1 (11.1)
**Location of primary tumor, *n* (%)**		
Caput	21 (75)	6 (66.7)
Corpus	4 (14.3)	0 (0)
Cauda	2 (7.1)	1 (11.1)
Corpus, cauda	1 (3.6)	1 (11.1)
Caput, corpus, cauda	0 (0)	1 (11.1)
**Tumor stage, *n* (%)**		
IA	1 (3.6)	2 (2.22)
IB	3 (10.7)	1 (11.1)
IIA	2 (7.1)	1 (11.1)
IIB	12 (42.9)	3 (33.3)
III	10 (35.7)	2 (22.2)
**Extracapsular extension to LN, *n* (%)**		
Yes	13 (46.4)	1 (11.1)
No	15 (53.6)	8 (88.9)
**Resection type, *n* (%)**		
Whipple	19 (67.9)	4 (44.4)
Distal pancreatectomy	5 (17.9)	1 (11.2)
Total pancreatectomy	4 (14.2)	4 (44.4)
**Comorbidities, *n* (%)**		
Chronic pancreatitis	1 (3.6)	1 (11.1)
Pancreatic insufficiency	1 (3.6)	0 (0)
Diabetes mellitus type 2	7 (25)	3 (33.3)
Diabetes mellitus type 3	0 (0)	1 (11.1)
Postoperative diabetes	4 (14.3)	4 (44.4)
Cholecystolithiasis	1 (3.6)	0 (0)
Heterozygote for Factor V Leiden	1 (3.6)	0 (0)

Supplementary Table S4: Clinical characteristics of patients after neoadjuvant chemotherapy or upfront surgery. Samples from the TMA used to compare GITR expression between neoadjuvant chemotherapy and upfront surgery patients.

	**Neoadjuvant (*n* = 4)**
**Age, years, mean (St.Err)**	60 (7.7)
**Sex, *n* (%)**	
Male	1 (25)
Female	3 (75)
**Relapse, *n* (%)**	
Yes	1 (25)
No	3 (75)
**PFS if relapse, days, mean (St.Err)**	13 (0)
**Deceased, *n* (%)**	
Yes	0 (0)
No	4 (100)
**OS if deceased, days, mean (St.Err)**	N/A
**Location of primary tumor, *n* (%)**	
Caput	1 (25)
Corpus	1 (25)
Corpus and cauda	2 (50)
Cauda	0 (0)
**Tumor stage, *n* (%)**	
I	0 (0)
II	2 (50)
III	1 (25)
IV	1 (25)
**Extracapsular extension to LN, *n* (%)**	
Yes	3 (75)
No	1 (25)
**Resection type, *n* (%)**	
Whipple	0 (0)
Distal pancreatectomy	1 (25)
Total pancreatectomy	3 (75)
**Neoadjuvant CTx, *n* (%)**	
FOLFIRINOX	4 (100)
**Neoadjuvant CTx cycles, n, mean (St.Err)**	9 (1.5)
**Comorbidities, *n* (%)**	
Postoperative diabetes	3 (75)

Supplementary Table S5: Clinical characteristics of patients after neoadjuvant chemotherapy. Samples from surgical PDAC resection used for spatial transcriptomic analysis.

	Short-term survivors (*n* = 22)	Long-term survivors (*n* = 17)
**Age, years, mean (St.Err)**	73 (2.1)	73 (1.6)
**Sex, *n* (%)**		
Male	11 (50)	10 (58.8)
Female	11(50)	7 (41.2)
**PFS, days, mean (St.Err)**	198 (34.5)	791 (125.2)
**OS, days, mean (St.Err)**	198 (22.9)	1708 (148)
**Precursor lesion, *n* (%)**		
IPMN	2 (9.1)	2 (11.8)
PanIN low grade	2 (9.1)	1 (5.9)
PanIN high grade	15 (68.2)	10 (58.8)
NA	3 (13.6)	4 (23.5)
**Location of primary tumor, *n* (%)**		
Caput	16 (72.7)	16 (94.1)
Corpus	2 (9.1)	1 (5.9)
Cauda	3 (13.6)	0 (0)
Corpus, cauda	1 (4.5)	0 (0)
**Tumor stage, *n* (%)**		
IA	2 (9.1)	0 (0)
IB	1 (4.5)	4 (23.5)
IIA	1 (4.5)	1 (5.9)
IIB	7 (31.8)	9 (52.9)
III	11 (50)	3 (17.6)
**Extracapsular extension to LN, *n* (%)**		
Yes	13 (59.1)	1 (5.9)
No	9 (40.9)	16 (94.1)
**Neoadjuvant chemotherapy**	0 (0)	3 (17.6)
**Resection type, *n* (%)**		
Whipple	13 (59.1)	14 (82.4)
Distal pancreatectomy	5 (22.7)	0 (0)
Total pancreatectomy	4 (18.2)	3 (17.6)
**Comorbidities, *n* (%)**		
Chronic pancreatitis	0 (0)	3 (17.6)
Pancreatic insufficiency	0 (0)	1 (5.9)
Diabetes mellitus type 2	5 (22.7)	6 (35.3)
Postoperative diabetes	4 (18.2)	4 (23.5)
Cholecystolithiasis	1 (4.5)	1 (5.9)
Cholecystectomy	0 (0)	1 (5.9)

Supplementary Table S6: Patient clinical characteristics of short-term and long-term survivors. Samples from the TMA used to compare GITR expression between short-term and long-term survivors.
